# N-terminus-independent activation of c-Src via binding to a tetraspan(in) TM4SF5 in hepatocellular carcinoma is abolished by the TM4SF5 C-terminal peptide application

**DOI:** 10.7150/thno.58739

**Published:** 2021-07-06

**Authors:** Haeng Eun Song, Yoonji Lee, Eunmi Kim, Chang Yun Cho, Oisun Jung, Doohyung Lee, Eun Goo Lee, Seo Hee Nam, Minkyung Kang, Stephani Joy Y. Macalino, Ji Eon Kim, Jae Woo Jung, Sung Won Kwon, Sun Choi, Jung Weon Lee

**Affiliations:** 1 Department of Pharmacy, College of Pharmacy, Seoul National University, Seoul 08826, Republic of Korea; 2 Research Institute of Pharmaceutical Sciences, Seoul National University, Seoul 08826, Republic of Korea; 3 Global AI Drug Discovery Center, College of Pharmacy and Graduate School of Pharmaceutical Sciences, Ewha Womans University, Seoul 03760, Republic of Korea; 4 College of Pharmacy, Chung-Ang University, Seoul 06974, Republic of Korea; 5 Interdisciplinary Program in Genetic Engineering, Seoul National University, Seoul 08826, Republic of Korea.

**Keywords:** c-Src, metastasis, protein-protein interaction, PTPIB, TM4SF5

## Abstract

Active c-Src non-receptor tyrosine kinase localizes to the plasma membrane via N-terminal lipid modification. Membranous c-Src causes cancer initiation and progression. Even though transmembrane 4 L six family member 5 (TM4SF5), a tetraspan(in), can be involved in this mechanism, the molecular and structural influence of TM4SF5 on c-Src remains unknown.

**Methods:** Here, we investigated molecular and structural details by which TM4SF5 regulated c-Src devoid of its N-terminus and how cell-penetrating peptides were able to interrupt c-Src activation via interference of c-Src-TM4SF5 interaction in hepatocellular carcinoma models.

**Results:** The TM4SF5 C-terminus efficiently bound the c-Src SH1 kinase domain, efficiently to the inactively-closed form. The complex involved protein tyrosine phosphatase 1B able to dephosphorylate Tyr530. The c-Src SH1 domain alone, even in a closed form, bound TM4SF5 to cause c-Src Tyr419 and FAK Y861 phosphorylation. Homology modeling and molecular dynamics simulation studies predicted the directly interfacing residues, which were further validated by mutational studies. Cell penetration of TM4SF5 C-terminal peptides blocked the interaction of TM4SF5 with c-Src and prevented c-Src-dependent tumor initiation and progression *in vivo*.

**Conclusions:** Collectively, these data demonstrate that binding of the TM4SF5 C-terminus to the kinase domain of inactive c-Src leads to its activation. Because this binding can be abolished by cell-penetrating peptides containing the TM4SF5 C-terminus, targeting this direct interaction may be an effective strategy for developing therapeutics that block the development and progression of hepatocellular carcinoma.

## Introduction

The c-Src proto-oncogene, non-receptor tyrosine kinase is overexpressed and activated in a diversity of cancer types 1. c-Src plays key roles in the regulation of cellular growth, division, adhesion, and motility 2. Thus, exploring how and which signaling molecules can affect c-Src activity may lead to the development of therapeutic agents against c-Src-mediated cellular functions 3. c-Src consists of the N-terminal myristoyl group attached to the Src homology (SH) 4 domain, SH3 domain, SH2 domain, proline-rich SH2 kinase linker region, and the SH1 or kinase domain 4. Regulation of c-Src occurs through inhibitory intramolecular interactions between the SH3 domain and the polyproline II helix formed by the SH2-kinase linker, as well as between the SH2 domain and Tyr530 in the C-terminal regulatory tail 2. Activation of c-Src is achieved when the SH3 domain is displaced from the SH2 linker by interacting with other proteins 5. Dephosphorylation of Tyr530 opens the inhibitory, or closed, conformation of c-Src, thereby activating the tyrosine kinase activity 6. Phosphorylation of Tyr419 in the activation loop essentially locks the kinase into its catalysis-ready conformation 7. Once activated further, c-Src translocates from the perinuclear regions to the plasma membrane via RhoA family-mediated actin organization 8. N-terminal myristoylation of c-Src can regulate trafficking to the cellular periphery, which determines its activity 9. Although many studies have focused on c-Src activation to reveal the mechanism of how the inhibitory intramolecular interactions occur, it remains unclear how diverse molecules, such as upstream effectors, regulate the conformation and activity of c-Src.

Previous studies have identified a few binding partners for c-Src, including focal adhesion kinase (FAK) 10 and platelet-derived growth factor receptor (PDGFR) 11, which bind SH2; negative regulatory factor (Nef) and sex-lethal interactor (Sin), which bind SH3 5; and p130Cas (Crk-associated substrate), which binds SH2 and SH3 12. These interactions with the SH2 and SH3 domains have been established to lead to c-Src activation. However, the protein that binds to pY530 in c-Src to positively regulate its activity regulation has not been known. Meanwhile, there are c-Src binders that inactivate c-Src, such as DOC-2/DAB2 (differentially expressed in ovarian carcinoma-2/disabled-2) 13, C-terminal Src kinase (CSK) 14, E3 ubiquitin‑ligase Cbl 15, 16, and Cullin‑5 17. In addition, c-Src activity was negatively regulated by Csk-binding protein (Cbp) in NIH3T3 cells 18, by cooperative roles of Cbp and Caveolin-1 19, and by Connexin43 in C6 glioma cells 20. The transactivator of transcription (TAT) cell-penetrating sequence fused to Connexin43 (TAT-Cx43_266-283_) has been shown to inhibit c-Src activity 21. The SH3 domain in c-Src interacts with the first proline-rich domain (amino acid 619- 627) of DOC-2/DAB2, leading to inhibition of c-Src Tyr416 phosphorylation. The edge of the active site of CSK interacts and phosphorylates Tyr530 on the C-terminal tails of c-Src. A binding partner for the c-Src SH1 domain and the C-terminal regulatory segment with Tyr530 may indeed affect c-Src conformation and activity.

Transmembrane 4 L six family member 5 (TM4SF5) is involved in tumorigenesis and tumor progression 22. TM4SF5 is a membrane glycoprotein with four transmembrane domains, two extracellular loops (SEL for short and LEL for long extracellular loop), an intracellular loop (ICL), as well as N-terminal and C-terminal tails that are located in the cytosol 23. Whereas the ICL of TM4SF5 binds the F1 lobe of the four-point-one, ezrin, radixin, moesin (FERM) domain in FAK for cell adhesion-dependent directional migration 24, the C-terminus of TM4SF5 supports c-Src-dependent invasive protrusions 25. However, it remains largely unknown how TM4SF5 regulates c-Src at the molecular and structural levels.

Here, we examined the mechanistic and biological significance of the interaction between the membrane protein TM4SF5 and c-Src. We found that the C-terminus of TM4SF5 bound to the SH1 domain of closed, inactive c-Src more strongly than open, active c-Src. Protein tyrosine phosphatase 1B (PTP1B) was additionally recruited for dephosphorylation at c-Src Tyr530. Thus, the TM4SF5 C-terminus activated the N-terminus-deficient c-Src by binding to its SH1 kinase domain. Computational simulation was performed to further explore the fundamental principles of how their structural organization and movements affect their binding to each other and ultimately govern their functions. Further, a unique interaction between the c-Src SH1 domain and TM4SF5 C-terminus was targeted by cell-penetrating peptides (CPPs) containing the TM4SF5 C-terminus during carcinogenesis and progression.

## Methods

**Cells:** Human hepatocellular carcinoma cells expressing TM4SF5-null control (SNU398, SNU449, SNU761 parental, SNU449_Cp_ [a pooled control clone], and SNU761-mock), TM4SF5_WT_ (NM-003963, 197 amino acids), SNU449_Tp_ or SNU449_T7_ [a pooled or single cell-driven clone with ectopic TM4SF5 expression, respectively], SNU761-TM4SF5_WT_ or mutant SNU761-TM4SF5_ΔICL19_ [a mutant in which the intracellular domain from amino acids 71 to 89 is deleted], and TM4SF5_ΔC_ [a mutant in which the C-terminus from amino acids 187 to 197 is deleted]) have been described previously 25. PLC/PRF/5 hepatoma cell line endogenously expressing TM4SF5 was purchased from Korea Cell Bank (Seoul National University, Seoul, Korea). HEK293FT cells and endogenous TM4SF5-expressing Huh7 and HepG2 cells were maintained in DMEM-H (WelGene, Daegu, Republic of Korea) containing 10% FBS and 1% penicillin/streptomycin (GenDEPOT, Barker, TX, USA) at 37°C in 5% CO_2_. Cells were routinely monitored for mycoplasma contamination.

**Peptides and DNA mutagenesis:** CPPs for TAT-Cscram (TCsr, 2703.1 Da), TAT-Cter (TC, 2703.1 Da), TAT-Caax-Cter (TcxC, 3149.8 Da), Antp-Caax-Cscram (3960.4 Da), or Antp-Caax-Cter (3960.4 Da) were synthesized and purchased (Anygen, Gwangju, Republic of Korea). The sequences of TAT and Antennapedia (Antp) are RKKRRQRRRP and RQIKIWFQNRRMKWKK, respectively, and the sequence of the TM4SF5 C-terminus is ^187^GDCRKKQDTPH^197^. The sequence of the Caax motif is CVIM, and it was conjugated between the TAT and TM4SF5 C-terminal sequences to avoid structural interference of the short TM4SF5 C-terminus, presumably without any secondary structure, although the Caax sequence is conjugated to the end of K-Ras for the cell-penetrating effects 26. Peptides were maintained at 1 mM for stock solutions in PBS and then diluted into culture media containing 10% FBS prior to treating the cells at different concentrations or conditions. c-Src point mutations (Y419F, Y530F, Y419F/Y530F, K298M, K298M/Y530F, K391, Q531E/P532E/G533I, or Y419F/Q531E/P532E/G533I, D454A, R460A, D454A/G459P) were engineered with *pfu* polymerase (Agilent-Stratagene, Santa Clara, CA, USA) and confirmed by direct sequence analyses. TM4SF5 mutants were also generated and confirmed by direct sequence analysis for C118A/C145A, D188A, C189A, and R190A. The ΔICL19 TM4SF5 mutation was described previously 23.

**Transfection of plasmids:** SNU449 or SNU761 parental or stable cells transfected with mock or TM4SF5-expressing plasmids were further transiently transfected using Lipofectamine Plus^TM^ (Thermo Fisher Scientific, Waltham, MA, USA) or electroporated with different plasmids for 48 h before whole cell lysates were collected. The plasmids included mock, STrEP-tagged TM4SF5_WT_, STrEP-tagged TM4SF5_C189A_ mutant, STrEP-tagged TM4SF5_C118A/C145A_ mutant, STrEP-tagged TM4SF5_D188A_ mutant, STrEP-tagged TM4SF5_R190A_ mutant, FLAG-tagged TM4SF5, c-Src WT, c-Src-SH432, c-Src-SH321, c-Src-SH1, c-Src_1-397_, c-Src-SH1_Y419F_, c-Src-SH1_Y530F_, c-Src-SH1_Y419F/Y530F_, c-Src-SH1_K298M_-HA, c-Src-SH1_K298M/Y530F_-HA, c-Src_D454A_-HA, c-Src_R460A_-HA, c-Src_D454A/G459P_-HA, c-Src_250-450_, c-Src_250-450-Y419F_, c-Src-SH321_Y419F_, c-Src-SH321_Y419F/Y530F_, c-Src-SH321_Q531E/P532E/G533I_, c-Src-SH321_Y419F/Q531E/P532E/G533I_, or PTP1B. siRNA for a control sequence (siControl) or PTP1B (siPTP1B) (Santa Cruz Biotechnology, Santa Cruz, CA, USA) were transiently transfected for 48 h.

**Cell lysate preparation and western blots:** Whole cell lysates were harvested from subconfluent cells cultured in normal media containing 10% FBS (Thermo Fisher Scientific) or transiently transfected with plasmids for 48 h using a lysis buffer containing 1% Brij58, 150 mM NaCl, 20 mM HEPES (pH 7.4), 2mM MgCl_2_, 2 mM CaCl_2_, and protease and phosphatase inhibitors. Then the lysates were normalized and immunoblotted using primary antibodies against pY397FAK, c-Src (ab39546), pS10p27^Kip1^ (ab62364) [Abcam, Cambridge, UK], pY577FAK (sc-16665-R), pY861FAK (sc-16663-R), pY925FAK (sc-11766-R), pY118paxillin (sc-101774), paxillin (sc-5574), PTP1B (sc-1718), c-Src (sc-8056, clone H-12), α-tubulin (sc-5286) [Santa Cruz Biotechnology], α-smooth muscle actin (A2547, Sigma-Aldrich, St. Louis, MO, USA), pY419c-Src (#2101), pY530c-Src (#2105) [Cell Signaling Technology, Danvers, MA, USA], FAK (clone 4.47, Cat #: 610088), pY397FAK (Cat #: 611723), p27^Kip1^(Cat #: 610242) [BD Transduction Laboratories, Bedford, MA, USA], or TM4SF5 27. Commercial antibodies were used at 1:1000 dilution in 1% BSA-containing PBS, and anti-TM4SF5 antibody was used at 1:10,000 ~ 20,000 dilution. Quantification of band intensity was performed from three independent blots using NIH Image J software and was normalized to loading controls.

**Co-immunoprecipitation:** Whole cell extracts were precipitated with anti-FLAG antibody (Cell Signaling Technology) or biotin-precoated beads (Sigma-Aldrich) in a cold room overnight prior to immunoblotting with the indicated antibodies. For co-immunoprecipitation of endogenous proteins, whole cell lysates of endogenous TM4SF5-expressing HepG2 cells were precipitated with either anti-TM4SF5 27 or c-Src (sc-8056, clone H-12) antibody prior to immunoblotting for FAK, c-Src, and TM4SF5. Immunoprecipitated proteins were boiled in 2× SDS-PAGE sample buffer before undergoing western blotting using standard protocols. Quantification of band intensity was performed as described above.

**Transwell migration or invasion assay:** Stable (TM4SF5-expressing) SNU449_Tp_ cells were infected with adenoviruses expressing the control HA vector (Ad-TA) or various forms of FAK, including WT, an N-terminal deletion mutant (FAKΔ [1-100]), a kinase dead K454R mutant (KAK-KD), or the FERM domain (FAK-FERM), in the absence or presence of 10 μM DMSO, PP2, PP3 (AG Scientifics, San Diego, CA, USA), or CPPs for 24 h. SNU761 cells stably expressing TM4SF5_WT_ were transiently transfected with control siRNA against a scrambled sequence (siControl) or PTP1B (siPTP1B) for 48 h before undergoing the Transwell migration assay for 4 h. Cells were treated with the CPPs before being loaded into the upper chambers. The cells were then analyzed for migration or invasion using Transwell chambers with 8-μm pores (Corning Inc., Corning, NY, USA), where the bottom chamber was filled with 1% BSA alone (in serum-free media) or normal culture media containing 10% FBS as explained previously 28. After incubation, the number of migrated cells from randomly saved images (n = 10) per condition was counted. The data are graphed as the mean ± standard deviation (SD) for each experimental condition.

**Invasive extracellular matrix (ECM) degradation analysis:** Cellular invasive degradation of the ECM was analyzed using Oregon Green® 488-conjugated-gelatin (10 μg/mL, Thermo Fisher Scientific) as described previously 28. Briefly, cells expressing FLAG (mock) or FLAG-TM4SF5 in the absence or presence of CPP treatment (10 μM) were placed on Oregon Green® 488-conjugated gelatin for 4 h prior to fixation with 3.7% formaldehyde in PBS for 10 min at room temperature [RT]); permeabilization with 5% Triton X-100 for 5 min at RT; blocking with 2% BSA in PBS for 30 min at RT; and then staining with phalloidin for 30 min at RT. Then the samples were examined for black spots, which indicate degradation of fluorescent gelatin, using a fluorescent microscope (BX51, Olympus, Tokyo, Japan). Relative ECM degradation by percent (%) was graphed as the mean ± SD (n=10) per condition.

**Indirect immunofluorescence:** Cells maintained under normal culture conditions on glass coverslips were treated with peptides for 1 day in the absence or presence of 10 μM TSAHC, a specific TM4SF5 inhibitor 29. The cells were incubated with either PBS (non-permeabilized) or 0.5% Triton X-100 in PBS (permeabilized) for 5 min at RT and then stained using an antibody against TAT (1:100 dilution, Cell Applications, Inc., San Diego, CA, USA). Cells treated with the CPPs were also fixed with 4% formaldehyde for 10 min at RT, incubated with 30 mM glycine for 30 min, permeabilized as described above, and then stained with antibodies against pY397FAK (Cat #: 611723, BD Transduction Laboratories, 1:500 dilution) or pY861FAK (sc-16663-R, Santa Cruz Biotechnology, 1:500 dilution). Phalloidin was also used to stain for F-actin. Immunofluorescent images were acquired on a confocal (Nikon eclipse Ti; Nikon, Melville, NY, USA) or a fluorescent microscope (BX51, Olympus, Japan).

**Mouse tumor xenograft:** Four-week-old female or male BALB/c-nu/nu mice were purchased from Orient Co. Ltd (Seungnam, Korea). All animal procedures were performed in accordance with the Seoul National University Laboratory Animal Maintenance Manual and with Institutional Review Board approval (SNU-130311-6-2, SNU-140423-11-7). Mice were housed in a specific pathogen-free room under controlled temperature and humidity. Viable SNU449_T7_ cells (5 × 10^6^) stably expressing TM4SF5 or PLC/PRF/5 cells (10^7^) endogenously expressing TM4SF5 were resuspended in sterile PBS and injected subcutaneously into 5-week-old mice (n = 5 or 6, respectively). Peptide treatment (0.074 or 0.222 nmol/g for TCsr and 0.064 or 0.190 nmol/g for TcxC for 7~8 days; 0.111 nmol/g for TCsr and 0.095 nmol/g for TcxC for 15 days) was initiated when the average tumor volume was approximately 100 mm^3^. Peptides or PP2/PP3 (3 mg/kg) were administered by intraperitoneal or subcutaneous injection, respectively, every day for 7~8 or 15 days. Body weight and tumor volume, which was determined as described previously 28, were measured daily.

***In vivo* lung metastasis analysis:** SNU449_T7_ cells (5 × 10^6^ cells/100 μL sterile PBS) were injected in the lateral tail vein of female BALB/c-nu/nu mice (n = 4). Two weeks later, peptides (0.037 or 0.185 nmol/g for TCsr and 0.032 or 0.158 nmol/g for TcxC) were intraperitoneally injected every other day for 2 weeks. All mice were sacrificed for lung analysis 2 weeks later. Metastatic colonies were counted macroscopically on the lung surface after staining with Bouin's solution (Sigma-Aldrich). In an orthotopic model, SNU449T_7_ cells stably transfected with tGFP-shScram or tGFP-shTM4SF5 (Origene Technologies, Inc) were orthotopically injected once into the livers of 6-week-old male BALB/c nude mice (5 × 10^5^ cells/mouse, n = 5). Four weeks later, the livers and lungs were analyzed for tumor formation.

**Statistical methods:** Data are presented as the mean ± SD and analyzed by ANOVA with Tukey's range-test or two-tailed unpaired Student's *t*-test to determine the statistical significance of differences observed between groups (GraphPad Prism, version 7, San Diego, CA, USA). *p*-values less than 0.05 were considered statistically significant.

**Homology modeling of TM4SF5:** The entire human TM4SF5 sequence was retrieved from the UniProtKB Database (UniProt id: O14894). An HHpred search 30 of this sequence suggested the X-ray crystal structure of CD81 (PDB id: 5TCX) 31 as a template; therefore, the sequence alignment deduced by HHpred was utilized as the initial sequence alignment. The sequence alignment was refined manually by comparing the secondary structure predicted by PSIPRED 4.0 32, transmembrane region predicted by TMHMM 33 and UniProt, and the disordered region predicted by DISOPRED 34. The homology model was then built using the RosettaCM modeling protocol 35. Among the 100 generated models, the best model was selected based on its scores, energy values, and appropriateness of the transmembrane (TM) region. The intracellular loop (ICL) was refined using RCD+, a fast loop modeling server 36. The highest scoring loop model was selected and merged to construct a whole sequence model.

**Protein-protein docking of the TM4SF5 C-terminus and the c-Src kinase domain:** For the direct docking of two different proteins, the fourth TM helix followed by the C-terminal tail of TM4SF5 was modeled using Modeller software 37 with the IVLCGIQLVNATIGVFCGDCRKKQDTPH sequence. (The underlined residues belong to the disordered C-terminal tail, and the remaining resides constitute the fourth TM helix). The resulting final helix model was docked to the X-ray crystal structure of inactive c-Src (PDB id: 2SRC) 38 using ClusPro protein-protein docking software 39. We manually investigated all final reported complexes (100 clusters) and selected the best model based on the following biological criteria: i) the helix binds to the SH1 domain of c-Src, ii) no steric hindrance to the possible membrane location, iii) no steric hindrance during the c-Src conformational change, and iv) the helix binds a region that is structurally different between the inactive and active forms of c-Src kinase. Then, the homology model of the whole TM4SF5 sequence was aligned to the final helix model of the docked complex structure.

**Simulations of molecular dynamics (MD) between TM4SF5 and the c-Src complex:** The TM regions of TM4SF5 were projected along the z-axis using the 5TCX.pdb (our template) entry in the Orientations of Proteins in Membranes (OPM) database. The input system of the TM4SF5 and c-Src complex structure was prepared using the Input Generator 40 and Membrane Builder 41 modules of CHARM-GUI (http://www.charmm-gui.org/). The TM regions of TM4SF5 were inserted into the 1-palmytoyl-2-oleoyl-sn-glycero-3-phosphatidylcholine (POPC) lipid bilayer, and the whole complex structure was solvated and neutralized with 0.15 M NaCl. The entire system, which was approximately 92.1×91.8×151.7 Å^3^ in size, contained one TM4SF5 chain, one c-Src chain, one cholesterol bound in TM4SF5, one ATP, and two Mg^2+^ ions bound to c-Src, 268 lipids, 92 sodium ions, 94 chloride ions, and 33,999 TIP3 water molecules. Then, the MD simulations were performed using Gromacs v.2018.4 42 with CHARMM36 force fields 43. After equilibration for 10 ns with an NVT ensemble, an additional equilibration step was performed with the NPT ensemble for 100 ns. The pressure was sustained at 1 bar using the Parrinello-Rahman method while the temperature was maintained at 300 K using the Nose-Hoover method. Non-bonded interactions had a 1.2 Å cutoff and 1.0 Å switching distance, and electrostatic interactions were calculated using the particle mesh Ewald (PME) method with a 1.2 Å cutoff. The bonds connected to hydrogen atoms were constrained using LINCS. Three independent production runs were performed for 1,000 ns with a time step of 2 fs. The trajectory analysis was performed using GROMACS v.2018.4 and VMD v.1.9.4.

**Sequence conservation analysis:** The sequences were retrieved from UniProtKB using the protein family name “L6 tetraspanin family” as a keyword. 'Fragment' sequences and those longer than 600 amino acids in length were removed. Multiple sequence alignment was conducted by MUSCLE (MUltiple Sequence Comparison by Log-Expectation) 44 and analyzed using Jalview 45. The sequence logo was created with WebLogo 46.

All molecular graphic figures were generated using PyMOL v.2.4.0 software (http://www.pymol.org), and the movies were prepared in VMD v.1.9.4 (http://www.ks.uiuc.edu/Research/vmd). Other graphs for the computational studies were generated with Grace-5.1.22/QtGrace v.0.2.6 (https://sourceforge.net/projects/qtgrace).

### Supplementary Information

Supplementary figures and movies can be found on the journal's web site.

## Results

### The c-Src kinase domain binds the TM4SF5 C-terminus

**Figure 1:**
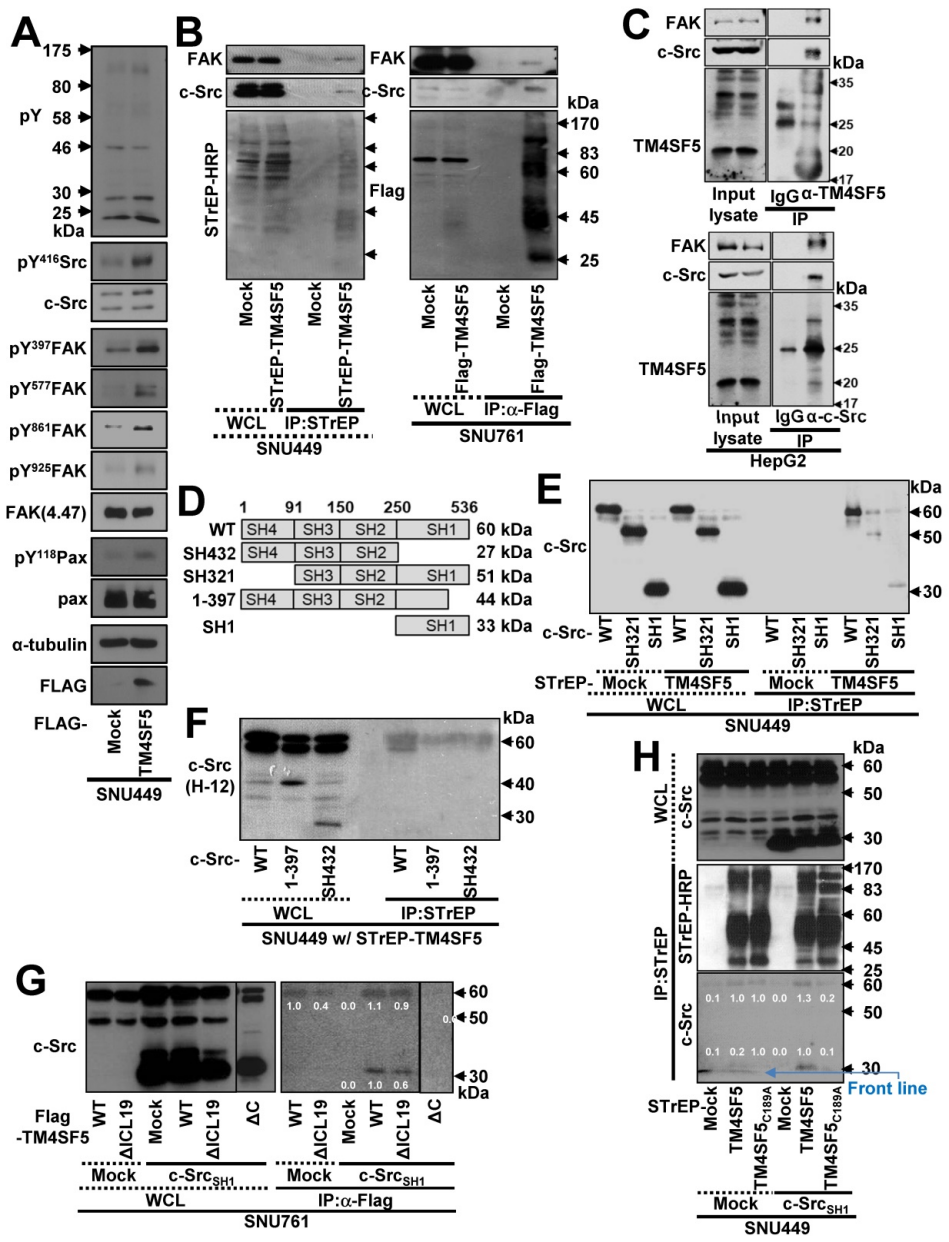
** The c-Src kinase domain binds the TM4SF5 C-terminus.** (A) Parental SNU449 cells null for TM4SF5 were transiently transfected with mock or TM4SF5-FLAG for 48 h, prior to whole cell lysate preparation and standard western blots for the indicated molecules. (B) SNU449 cells stably-transfected with STrEP-tagged empty vector (mock) or TM4SF5, and SNU761 cells stably-expressing either FLAG-tagged empty vector (mock) or FLAG-TM4SF5 were harvested for whole cell lysates (WCL). The lysates were pulled-down with streptavidin-beads or with sepharose beads coated with anti-Flag antibody. The proteins pulled-down and WCL were then immunoblotted for anti-FAK, anti-c-Src, anti-STrEP-HRP, or anti-FLAG tag. (C) HepG2 cell lysates were immunoprecipitated with normal IgG or anti-TM4SF5 or anti-c-Src antibody before immunoblots for FAK, c-Src, and TM4SF5. (D) Schematic presentation of the human c-Src constructs. (E-F) SNU449 cells transfected with STrEP-mock, STrEP-TM4SF5 and either pcDNA3-c-Src WT, SH321, or SH1 construct (E) or either pcDNA3-c-Src WT, SH432, or 1-397 construct (F) for 48 h were harvested. The whole cell lysates (WCL) were precipitated with streptavidin beads prior to immunoblotting for c-Src. (G) SNU761 cells stably expressing TM4SF5_WT_, TM4SF5_△ICL19_ (△ICL19), or TM4SF5_△C_ (△C) deletion mutant were transiently transfected with either c-Src WT or the SH1 construct, were harvested for WCL. The lysates were immunoprecipitated with anti-FLAG beads prior to immunoblotting for c-Src. (H) SNU449 cells transiently transfected with STrEP-mock, -TM4SF5_WT_, or -TM4SF5_C189A_ mutant together with either pcDNA3 mock or c-Src SH1 construct were harvested for WCL, and the lysates were precipitated with streptavidin beads, prior to immunoblotting in parallel with the WCL. Quantitative comparison ratios of band intensities were calculated by measurement of band intensities using Image J and their normalization to those of loading controls. The data shown represent three independent experiments.

Ectopic overexpression of TM4SF5 activated cell adhesion-related signaling molecules, including FAK, c-Src, and paxillin (Figure 1A). Furthermore, human hepatic tumor tissues exhibited FAK activation that coincided with TM4SF5 overexpression (Figure S1A). As TM4SF5 is known to bind and activate FAK 24 and c-Src family kinases (SFKs) 25, we confirmed the binding of endogenous FAK and c-Src to exogenous TM4SF5 in hepatic cancer cells (Figure 1B). Furthermore, immunoprecipitation of endogenous TM4SF5 or c-Src in HepG2 cells co-precipitated with TM4SF5, c-Src, and FAK (Figure 1C). We further explored how the interaction between TM4SF5 and c-Src might play roles in cellular migration signaling. Overexpression of FAK_WT_ in TM4SF5-expressing SNU449_Tp_ cells caused a dramatic increase in migration, compared with control mock cells (Figure S1B). However, cells expressing a FAK kinase dead mutant (KD with R454 mutation) or the N-terminus of FAK (1-100 amino acids) still exhibited more migration than mock-infected cells. The residual migration of FAK-KD cells was further abolished by the specific c-Src inhibitor, PP2, but not by a control reagent, PP3 (Figure S1C). Therefore, cell migration could be exclusively controlled by c-Src or FAK activity.

To determine which region of c-Src interacts with TM4SF5, we constructed several c-Src constructs (Figure 1D) and performed co-immunoprecipitation assays. SNU449 cells were transiently co-transfected with STrEP-mock or -TM4SF5 and c-Src WT, SH321, or SH1 constructs. TM4SF5 co-immunoprecipitated with each c-Src construct, suggesting that the binding domain included the c-Src SH1 domain (Figure 1E). Consistent with this, the domain consisting of amino acids 1 to 397 (44 kDa), which does not include the complete SH1 domain, and the SH432 domain (27 kDa) did not bind to TM4SF5, unlike exogenous and endogenous c-Src WT (Figure 1F).

TM4SF5_WT_ bound to endogenous c-Src WT (60 kDa) or an exogenous SH1 (33 kDa) domain (Figure 1G, lanes 7 and 10). Further, FLAG-TM4SF5_WT_ and TM4SF5_ΔICL19_, in which 19 amino acids were deleted from the ICL (aa 71 to 89, 18.9 kDa), bound to both endogenous c-Src WT and an exogenous SH1 domain (Figure 1G, lanes 8 and 11). However, TM4SF5_ΔC_, which lacked the C-terminus (aa 189 to 197, 19.7 kDa), did not bind to either endogenous c-Src WT or exogenous c-Src SH1 domain (Figure 1G, lane 12). These results suggest that the C-terminus but not the ICL of TM4SF5 bound to the SH1 domain of c-Src. Consistent with this, a point mutation in the C-terminus of TM4SF5 (C189A) led to loss of binding to endogenous c-Src WT and an exogenous SH1 domain (Figure 1H, lane 6). Together, these observations demonstrate that the C-terminus of TM4SF5 interacts with the SH1 domain of c-Src.

### TM4SF5 preferentially binds inactively-closed c-Src forms

**Figure 2:**
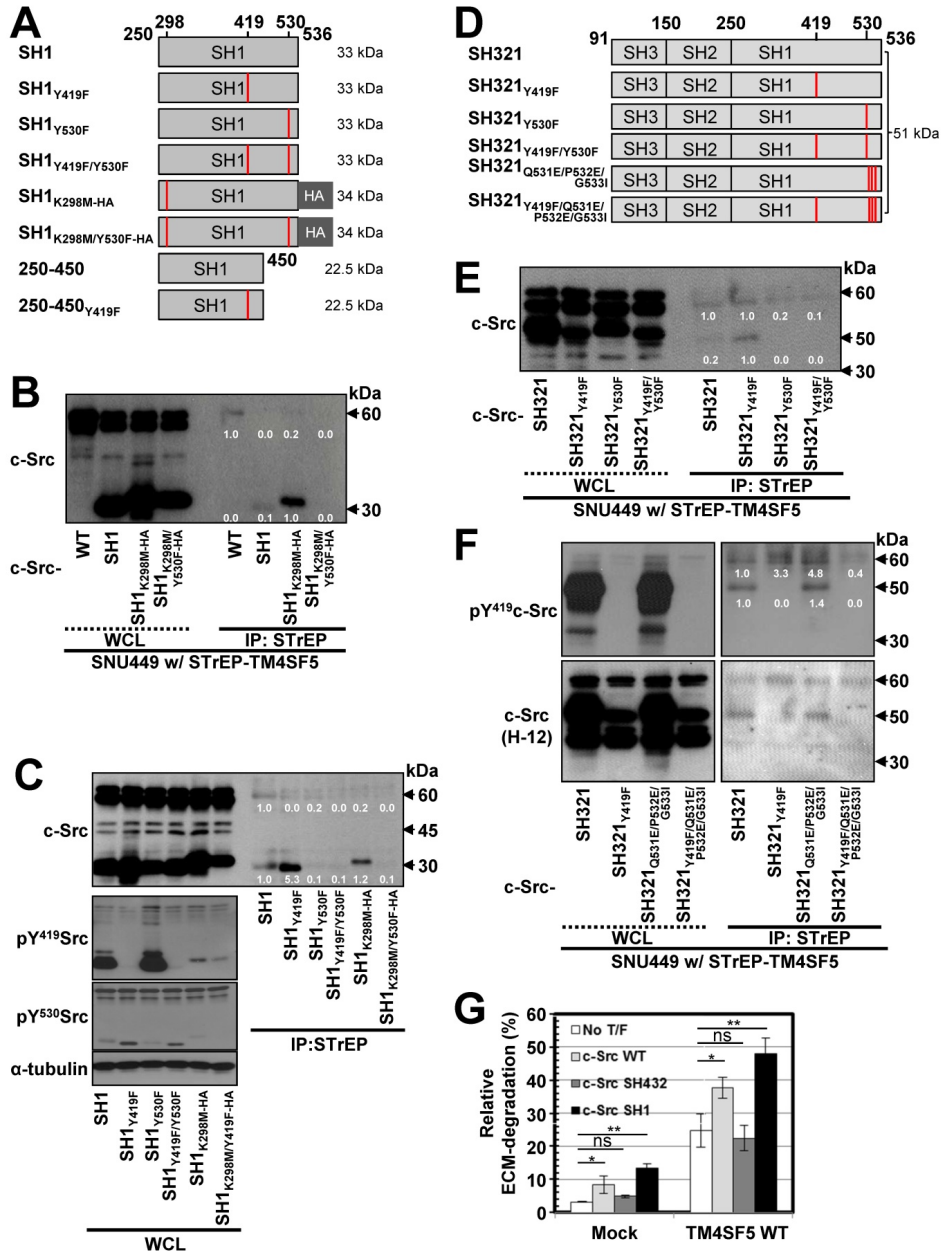
** TM4SF5 preferentially binds inactively-closed c-Src forms.** (A) Schematic presentation of the human c-Src SH1 domain constructs. (B and C) SNU449 cells stably expressing STrEP-TM4SF5 transiently transfected with either c-Src construct were harvested for whole cell lysates (WCL), and the lysates were precipitated with streptavidin beads, prior to immunoblotting for c-Src. The various c-Src constructs included c-Src WT, SH1, SH1_K298M_-HA, and SH1_K298MY530F_-HA construct in (B), or SH1, SH1_Y419F_, SH1_Y530F_, SH1_Y419F/Y530F_, SH1_K298M_-HA, and SH1_K298M/Y530F_-HA constructs in (C). (D) Schematic presentation of the human c-Src SH321 domain constructs. (E and F) SNU449 cells transiently transfected with STrEP-TM4SF5_WT_ construct together with either c-Src SH321 construct were harvested and precipitated with streptavidin agarose beads, prior to immunoblotting for c-Src, or phospho-Y419 c-Src. The various c-Src SH321 constructs included c-Src SH321, SH321_Y419F_, SH321_Y530F_, SH321_Y419F/Y530F_ constructs in (E), or SH321, SH321_Y419F_, SH321_Q531E/P532E/G533I_, and SH321_Y419F/Q531E/P532E/G533I_ constructs in (F). (G) SNU761 cells stably expressing mock or TM4SF5 WT were transiently transfected without (No T/F) or with c-Src WT, c-Src SH432, or c-Src SH1 expression constructs for 48 h prior to invasive ECM degradation analysis for 4 h. Quantitative comparison ratios were calculated from the blots by measurement of band intensities using Image J and normalization of them to those of loading controls. The data shown represent three independent experiments.

To further understand the mechanism and phosphorylation status of c-Src binding to TM4SF5, we generated various point mutant constructs of the c-Src SH1 domain and evaluated their interactions through pull-down assays (Figure 2A). These constructs contained point mutations at the autophosphorylation residue (Y419), the inhibitory phosphorylation (Y530) residue that binds the SH2 domain, and/or a kinase disabling residue (K298M) 47. First, STrEP-tagged TM4SF5_WT_ was found to bind to c-Src WT and the SH1 domain. However, this binding was found to be stronger towards exogenous SH1_K298M_-HA or SH1_Y419F_, which are kinase-inactive. However, binding by the kinase-inactive mutants was lost with introduction of an additional mutation at Y530F (SH1_K298M/Y530F_-HA or SH1_Y419F/Y530F_, Figure 2B-C). Further, the exogenous c-Src SH1 domain with Y530F mutation did not bind to TM4SF5 (Figure 2C). Furthermore, binding of the SH1 domain to STrEP-TM4SF5 increased in the presence of the Y419F mutation that results in an inactive form of c-Src (Figure 2C). Thus, phosphorylation of c-Src at Y530 might be important for binding to TM4SF5.

We next examined the significance of the interaction between the c-Src SH2 domain and Y530 in TM4SF5/c-Src binding using the SH321 form (51 kDa) of c-Src. TM4SF5 bound endogenous c-Src, exogenous SH321, and SH321_Y419F_, which exhibited the strongest binding. However, TM4SF5 binding was lost with Y530F or Y419F/Y530F mutation in the SH321 domain (Figure 2D-E), similar to the SH1 domain. Our data for the SH1 and SH321 domains thus indicate that inactive c-Src in the closed form binds TM4SF5 more strongly. Further, we examined the binding of a closed and repressed c-Src construct by introducing mutations at the Q531, P532, G533 residues in the SH321 construct (Figure 2D). Mutation of these residues (i.e., Q528E/P529E/G530I) results in a constitutively closed state 48. This closed c-Src SH321_Q528E/P529E/G530I_ form bound to TM4SF5, similarly to the SH321 construct; however, addition of the Y419F mutation abolished this binding (Figure 2F, lanes 5, 7, and 8). Further, we found that TM4SF5 expression increased invasive ECM degradation, which was further enhanced by additional transfection of c-Src WT or c-Src SH1 forms that bind TM4SF5, but not by c-Src SH432, which does not bind to TM4SF5 (Figure 2G, S2A). Together, these results suggest that c-Src phosphorylation at Y530, in addition to a closed conformation, is involved in TM4SF5 binding to mediate cellular invasion.

### Interaction between TM4SF5 and inactive c-Src is regulated by PTP1B

**Figure 3:**
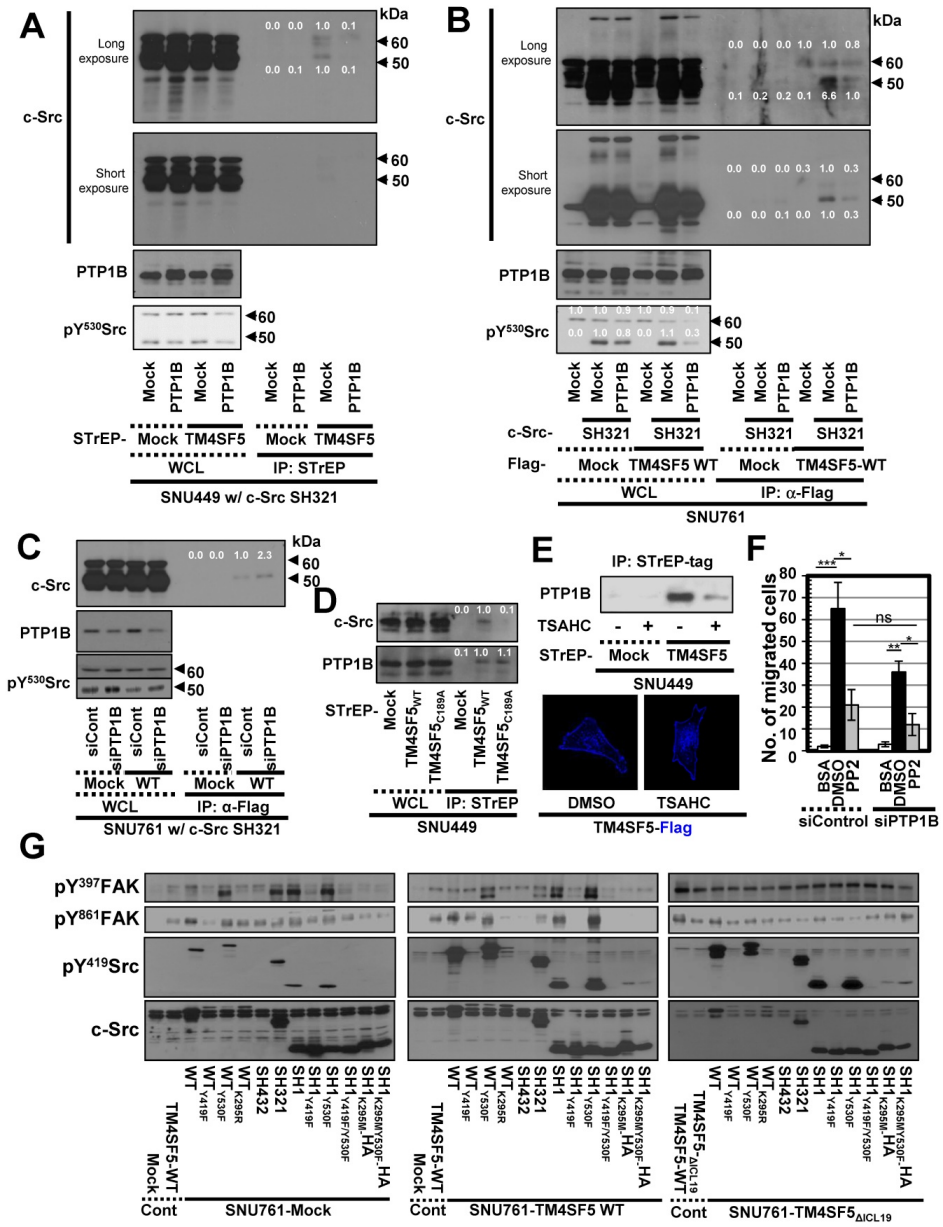
** Triple-complex between TM4SF5, inactive c-Src, and PTP1B leads to c-Src activation.** (A and B) SNU449 cells transiently transfected with the c-Src SH321 construct together with the indicated constructs were harvested (A). SNU761 cells stably expressing mock or FLAG-TM4SF5 were transiently transfected with the indicated constructs were harvested (B). The whole cell lysates (WCL) were precipitated with streptavidin beads, prior to immunoblotting. (C) SNU761 cells stably expressing mock or FLAG-TM4SF5_WT_ (WT) were transiently transfected with the indicated construct and siRNAs against a scrambled sequence (siCont) or a sequence in PTP1B (siPTP1B). Immunoprecipitation from the WCL using anti-Flag-precoated sepharose beads was performed, before immunoblots. (D) SNU449 cells transfected with the indicated plasmids were processed for precipitation using streptavidin beads, prior to immunoblotting. (E) Stable SNU449 cells were treated with DMSO (-) or TSAHC (20 μM, +) for 24 h, before harvest, precipitation using streptavidin beads, and immunoblotting. Another cell set with TM4SF5-Flag and treated with DMSO or TSAHC as above was processed for immunofluorescence analysis. (F) SNU761 cells stably expressing TM4SF5_WT_ were transiently transfected with the indicated siRNAs for 48 h, prior to transwell migration assay. The mean ± SD were presented in the graph. ANOVA with Tukey's range-test or two-tailed unpaired Student's *t*-test was done to determine the significance. NS depicts non-significance. *, **, and *** depict *p* value less than 0.05, 0.01, and 0.005, respectively. (G) SNU761 cells stably expressing the indicated form were transiently transfected with either c-Src expression construct for 48 h were processed to immunoblotting. For comparison of their levels, control (Cont) lysates prepared from mock, TM4SF5_WT_, or TM4SF5_△ICL19_ cells without further c-Src construct transfection were also immunoblotted in parallels. Quantitative comparison ratios were calculated from the blots by measurement of band intensities using Image J and normalization of them to those of loading controls. The data shown represent three independent experiments.

c-Src activity can be regulated by several protein tyrosine phosphatases (PTPs), such as PTP1B, to dephosphorylate Y530 and regulate its kinase activity 49. Therefore, we transfected SNU449 cells with control, STrEP-TM4SF5, and/or PTP1B plasmids together with c-Src SH321 constructs. As expected, STrEP-TM4SF5 bound to endogenous c-Src (60 kDa) and the exogenous c-Src SH321 domain (51 kDa). Additional transfection of PTP1B, following a reduction in pY^530^c-Src, abrogated this binding to the exogenous SH321 form (Figure 3A). In another cell line, SNU761, transfection of PTP1B decreased the binding between FLAG-TM4SF5 and 51 kDa c-Src SH321 (Figure 3B). Interestingly, PTP1B transfection into TM4SF5-positive cells decreased Tyr530 phosphorylation more in the presence of endogenous c-Src WT and exogenous SH321 c-Src forms than in TM4SF5-negative cells (Figure 3B, bottom), indicating a role for TM4SF5-dependent PTP1B activity in Tyr530 dephosphorylation.

These observations using different expression vectors further confirms that tags in the TM4SF5 constructs do not affect binding. Meanwhile, transient PTP1B suppression using specific siRNA increased binding between FLAG-TM4SF5 and c-Src SH321 (Figure 3C). Therefore, the association between TM4SF5 and c-Src can be dependent on the phosphorylation of c-Src Tyr530 and regulated by PTP1B. Interestingly, whereas TM4SF5_WT_ could bind both c-Src and PTP1B, the TM4SF5_C189A_ mutant bound PTP1B but not c-Src (Figure 3D). Further, we tested whether the interaction between TM4SF5 and PTP1B could be targeted using a TM4SF5 inhibitor, 4-(*p*-toluenesulfonylamido)-4-hydroxychalcone (TSAHC) 29. Although TSAHC-treated TM4SF5-positive cells still showed membrane localization of TM4SF5 (Figure 3E, images), PTP1B binding to TM4SF5 was reduced substantially following treatment with TSAHC (Figure 3E, blots). Since TSAHC binds 50 and affects the LEL of TM4SF5 29, these results indicate that the LEL domain is functionally linked to TM4SF5 C-terminus-mediated c-Src activation.

Next, we found that chemotactic migration of SNU761-TM4SF5 cells was abolished by PP2, a specific c-Src inhibitor. Once c-Src was inhibited, PTP1B suppression did not produce any further effects (Figure 3F and S2B). Therefore, these observations indicate that TM4SF5/c-Src binding with coordinated PTP1B activity is involved in TM4SF5-mediated migration. To further physiologically specify the signaling pathway for TM4SF5-dependent c-Src activation, several c-Src forms were introduced into SNU761 cells stably expressing mock, TM4SF5_WT_-FLAG, or TM4SF5_ΔICL19_-FLAG and then examined for phosphorylation events. Different c-Src constructs increased pY^419^ in the exogenous c-Src forms, which were higher in TM4SF5_WT_- and TM4SF5_ΔICL19_-expressing cells compared to mock control cells (Figure 3G). Interestingly, transfection of several c-Src constructs into SNU761-mock cells positively correlated with increased pY^397^FAK, but not pY^861^FAK, levels in parallel to basal (i.e., TM4SF5-independent) pY^419^c-Src levels (Figure 3G, left panel). However, when TM4SF5_WT_ cells were transfected with different c-Src forms, their activity varied depending on their interaction with TM4SF5_WT_ and correlated well with pY^861^FAK and pY^397^FAK levels (Figure 3G, middle panel). Alternatively, in cells expressing TM4SF5_ΔICL19_, transfection of different c-Src constructs increased pY^419^c-Src levels; however, this increase did not correlate to FAK phosphorylation (Figure 3G, right panel). Thus, it is likely that the interaction of TM4SF5 with c-Src regulates its ability to phosphorylate Y^861^ on FAK, also with being correlated with pY^397^FAK levels.

### Structural modeling identifies the key interfacing residues between TM4SF5 and c-Src

**Figure 4:**
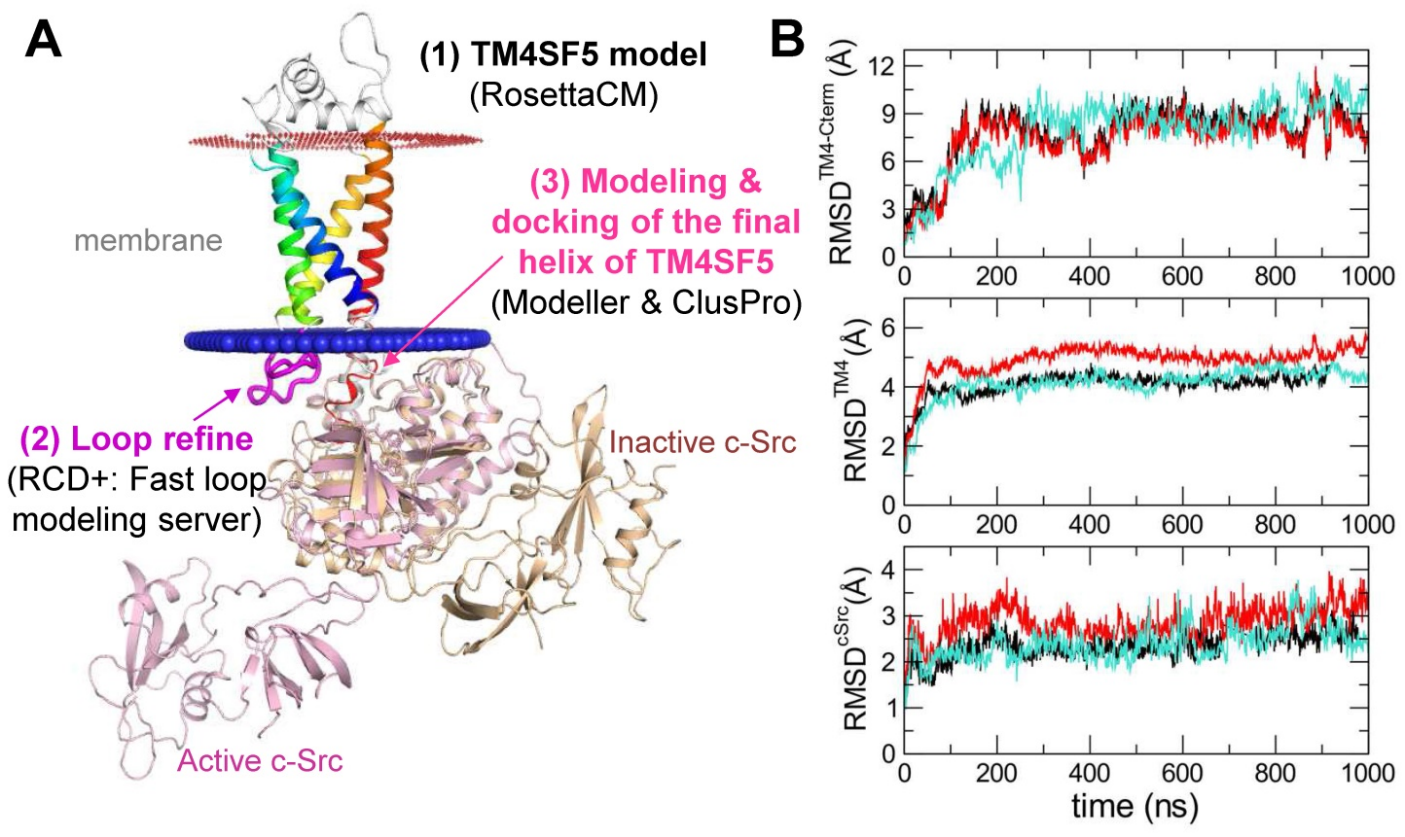
** Prediction of the complex structure of TM4SF5 and c-Src.** (A) Model generation process and the resulting complex structure. The transmembrane region of TM4SF5 is colored by blue-to-red (N-to-C termini). The loop regions are colored in white except the ICL in magenta where the loop refinement was conducted. The inactive c-Src structure in the docked complex is colored in light yellow. The active c-Src (pink) was aligned to the inactive c-Src structure to speculate the possible conformational change after the kinase activation. (B) The Root-Mean-Square-Deviation (RMSD) values for the C-terminal region of TM4SF5 (top), the transmembrane region of TM4SF5 (middle), and c-Src (bottom) during the MD simulation. The results from the three independent trajectories are colored in black, red, and cyan. Note that the C-terminal tail of TM4SF5 showed dramatic increase in their RMSDs, which means this region underwent drastic conformational change.

In order to identify the interfacing residues between TM4SF5 and c-Src at the molecular level, we performed structural modeling of their complex structures (Figure 4A). First, a homology model of TM4SF5 was constructed with RosettaCM 35 using the X-ray crystal structure of the genuine tetraspanin CD81 (pdb id: 5TCX) 31 as a template (Figure S3A-B). The long ICL region was refined by RCD+ 36. The final helix of TM4SF5 (the 4th transmembrane domain, TM4) with the long floppy C-terminal tail was modeled with Modeller 37 (Figure S3C) and docked to the SH1 kinase domain of c-Src with the ClusPro protein-protein docking program 39 (Figure S3D). The appropriate conformation of the TM4 connected to the C-terminal tail was selected based on several criteria (Figure S3E). Then, the full sequence model of TM4SF5 was aligned to the TM4 region docked to c-Src, thus completing the entire complex. This structure was further optimized by MD simulations in the explicit membrane and water system for a 1 microsecond production run.

**Figure 5:**
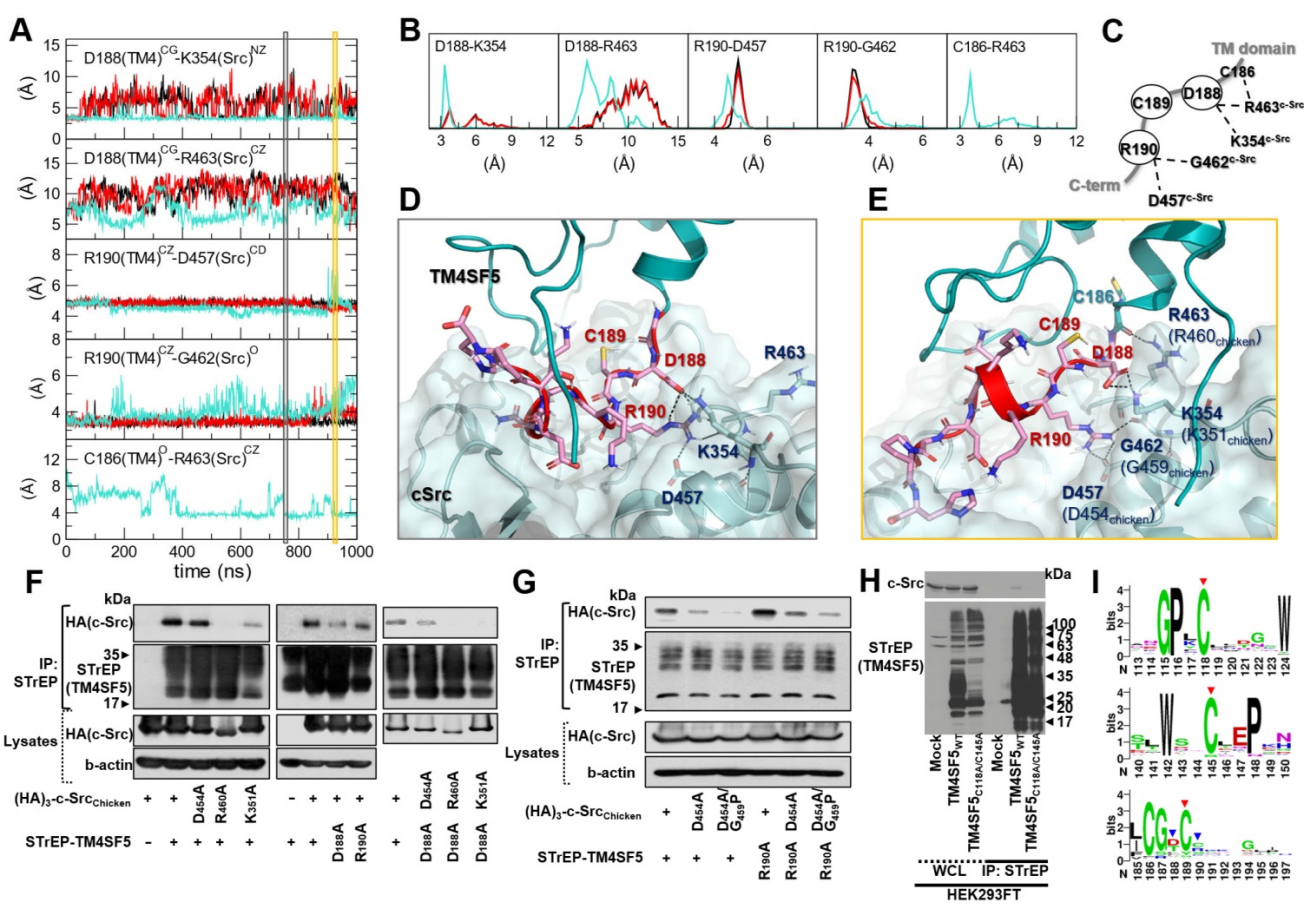
** Direct key interactions and dynamic hydrogen bonding network between the TM4SF5 C-terminus and the c-Src SH1 kinase domain.** (A) The time traces of the distances between the key residue pairs. The results from the three independent trajectories are colored in black, red, and cyan. (B) The histogram of the distances in (A). (C) Schematic diagram of the dynamic hydrogen bonding network observed during the simulation. (D and E) Detailed molecular interactions at the time point marked in the gray and yellow boxes in (A). The TM4SF5 transmembrane region is colored in teal, and the C-terminal tail residues are represented in sticks with their carbon atoms in pink. The interacting residues in c-Src are depicted in sticks with their carbon atoms in light blue. The molecular surface of c-Src is also displayed in light blue. The hydrogen bonding or ionic interactions are displayed in black dashed lines. (F and G) SNU449 cells were transiently transfected with indicated plasmids for 48 h, before whole cell lysate preparation and co-immunoprecipitation, before immunoblots. (H) HEK3293FT cells transfected with the indicated expression constructs were processed for co-precipitation and immunoblotting. The data shown represent three independent experiments. (I) Sequence conservation of the key residues. The larger sequence character size corresponds to the higher sequence conservation. The X-axis is for the residue number of human TM4SF5. The highly conserved cysteine residues in the mutational analysis are depicted with red arrows, and the residues involved in direct binding to c-Src are marked with blue arrow.

The C-terminal tail of TM4SF5 seems to be structurally disordered, i.e., highly flexible in our body (predicted as a disordered region by DISOPRED 34, data not shown). These highly flexible structural properties are difficult to capture using traditional structural determination techniques; however, they are believed to be a key component of cellular signaling and regulatory networks. By staying flexible, these regions have an advantage in interacting with other partner proteins because the floppy state allows them to respond faster than a rigid structure. By applying MD simulations, we could observe how this floppy tail interacts and fluctuates collectively to govern protein function (Movie S1). As expected, the C-terminal tail of TM4SF5 was highly mobile inside the binding cavity of c-Src (Figure 4B). Note that the RMSD values of the C-terminal tail of TM4SF5 increased dramatically during the simulation compared to the TM region of TM4SF5 and c-Src. Nevertheless, we were also able to observe several intermolecular interactions that were maintained at high probability (Movie S2). In TM4SF5, the D188 and R190 residues were predicted to be involved in binding to c-Src residues, such as K354, R463, and D457 (the residue numbers follow that of human c-Src). These residue pairs could form ionic/H-bond interactions in the significant portion during the simulation (Figure 5A-B), thus maintaining the association between TM4SF5 and c-Src. These interactions could be broken sometimes, but they immediately switched to make other neighboring interactions. D188^TM4SF5^ formed ionic interactions with K354^c-Src^ or R463^c-Src^ while R190^TM4SF5^ interacted with D457^c-Src^ or G452^c-Src^ (Figure 5C). These dynamic H-bond/polar networks (Figure 5D-E) seemed to be responsible for the binding mechanism of the floppy C-terminal tail of TM4SF5 at the binding site of c-Src.

To experimentally validate these interactions, we performed point mutation analysis and confirmed the importance of D188^TM4SF5^, K354^c-Src^ (K351 in chicken), and R463^c-Src^ (R460 in chicken) in the association between these two proteins (Figure 5F). In this test, however, we could not conclude that any binding occurs between R190^TM4SF5^ and D457^c-Src^ (D454 in chicken). This may be because the intermolecular contacts in the protein-protein interaction might be so dynamic that point mutation of one specific residue could sometimes be compensated by other possible interactions. Therefore, we speculate that if the interactions between D188^TM4SF5^ and its binding partners are strong, a single point mutation of R190^TM4SF5^ itself cannot significantly affect complex formation. In particular, D188A^TM4SF5^ and D457A^c-Src^ are not direct binding partners since cotransfection of each mutation construct still showed a binding similar to that of the WT proteins (see the third panel of Figure 5F). Interestingly, in our simulation, R190^TM4SF5^ interacted tightly with D457^c-Src^ (see the third panel of Figure 5A) and bound to the backbone of G462^c-Src^ (see the fourth panel of Figure 5A). Therefore, we tested D457A^c-Src^ (D454 in chicken) and G462P^c-Src^ (D459 in chicken) with a backbone conformation change using a glycine to proline mutation. We confirmed that the D457A^c-Src^ and D457A/G462P^c-Src^ (D454A and G459P in chicken) mutations impaired binding to TM4SF5_WT_ or R190A^TM4SF5^, although the R190A^TM4SF5^ mutant still bound to c-Src WT (Figure 5G).

In addition, we found that some of the highly conserved cysteine residues in TM4SF5 affect its binding to c-Src. As shown in Figure 1G, the C189A^TM4SF5^ mutation led to loss of binding to c-Src. A direct interaction was not observed between C189^TM4SF5^ and the c-Src residues during the simulation. However, this residue appears to be important for maintaining the appropriate conformation, especially since it is located between D188^TM4SF5^ and R190^TM4SF5^, which are responsible for the direct binding to c-Src. Further, C118 and C145 in the LEL domain of TM4SF5 were found to be critical for c-Src binding (Figure 5H), suggesting that the LEL is functionally linked to the cytosolic C-terminus of TM4SF5 for c-Src binding and therefore TM4SF5/c-Src-mediated cellular functions. Sequence conservation analysis of the protein family (L6_membrane protein, PF05805 accession code in Pfam 51) showed that many of the above-mentioned cysteine residues are conserved (Figure 5I, red arrows), reflecting their importance in the biological function and structural integration of this protein family. Meanwhile, D188 and R190, which are responsible for direct binding to c-Src. showed less conservation at the family level (Figure 5I, blue arrows). It is noteworthy that the reduced sequence conservation of these direct-binding residues reflects the uniqueness and selectivity of the ability of TM4SF5 to bind c-Src and regulate its activity. Therefore, the binding of TM4SF5 to c-Src involves structural integrity from the LEL (i.e., EC2) to the cytosolic C-terminus of TM4SF5 and direct interaction between this C-terminal region (including residue D188, C189, and R190) and the SH1 kinase domain of c-Src (including K354, D457, G462, and R463).

### Cell-penetrating TM4SF5 C-terminal peptides disrupt the interaction between TM4SF5 and c-Src

**Figure 6:**
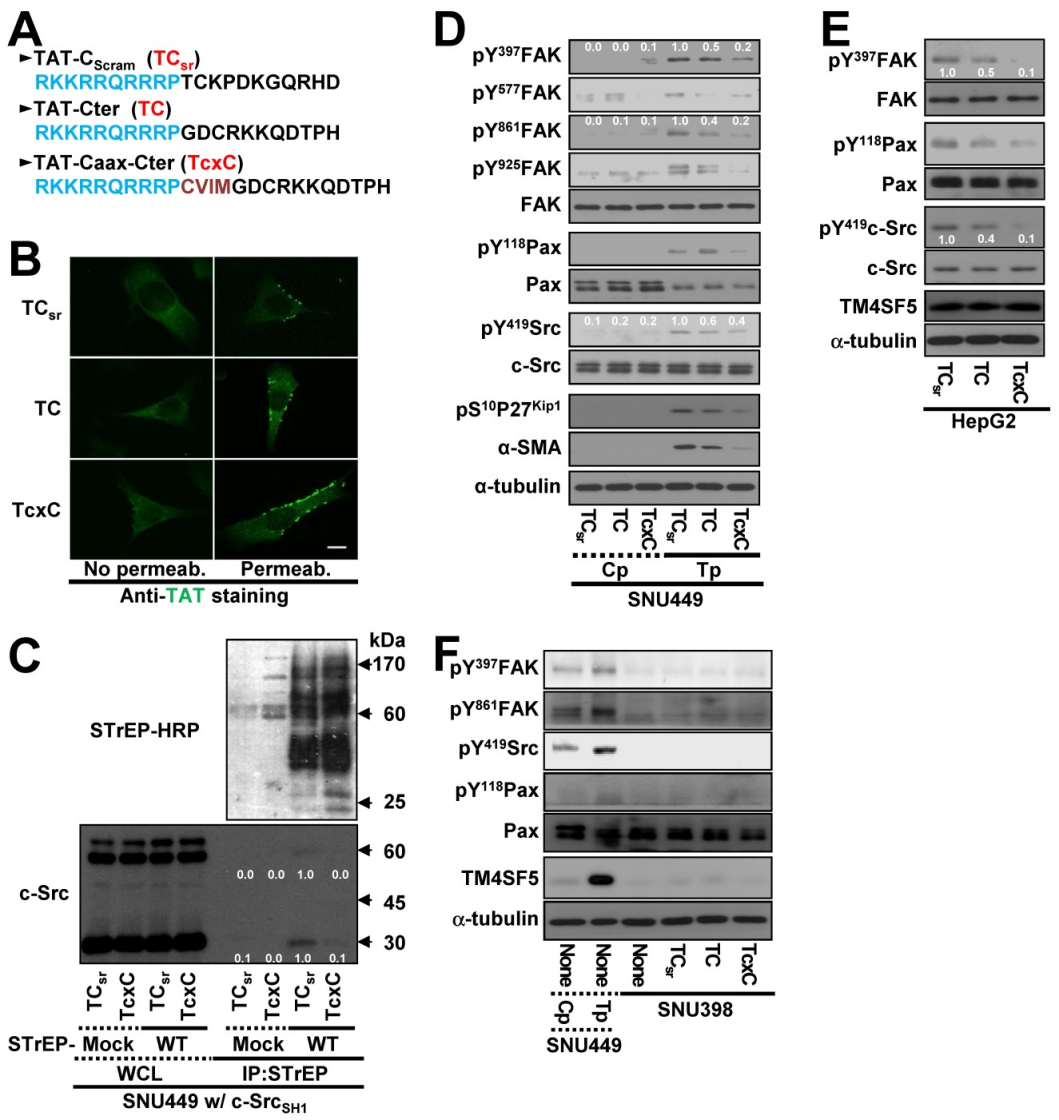
** Cell-penetrating TM4SF5 C-terminal peptides inhibit the TM4SF5 binding-mediated c-Src activation.** (A) The sequences of the CPPs containing TAT-conjugated with a control (i.e., scrambled) sequence (TC_sr_) and the TM4SF5 C-terminal sequence without (TAT-Cter, TC) or with a CVIM motif (TAT-Caax-Cter, TcxC). (B) SNU449 cells were treated with the CPPs at 10 μM, prior to performing indirect immunofluorescence using anti-TAT (green). Incubation of the cells with the primary antibody was performed without (No Permeab.) or with pre-permeabilization (Permeab.). (C) SNU449 cells transiently transfected with STrEP-mock or TM4SF5_WT_ (WT) together with pcDNA3 c-Src SH1 construct were treated with the CPPs (10 μM) for 24 h and then harvested for the WCL. The lysates were precipitated with streptavidin beads prior to immunoblotting for c-Src and Streptavidin (STrEP-HRP). (D to F) Control SNU449_Cp_ (Cp) or ectopically TM4SF5-expressing SNU449_Tp_ (Tp) cells (D), HepG2 cells endogenously expressing TM4SF5 (e), or TM4SF5-null SNU398 cells (F) were treated with the CPPs (10 μM), before harvests of WCL and immunoblotting for the indicated molecules. The lysates from SNU449_Cp_ (Cp) and SNU449_Tp_ (Tp) cells were used as a negative or a positive control of TM4SF5 expression. Quantitative comparison ratios were calculated from the blots by measurement of band intensities using Image J and normalization of them to those of loading controls. The data shown represent three independent experiments.

Because the interaction between the TM4SF5 C-terminus and c-Src SH1 domain appeared to be important for c-Src activation, we hypothesized that a CPP containing the TM4SF5 C-terminal sequence might disrupt this binding and lead to other downstream effects. CPPs were designed with a fragment of HIV-TAT protein ([TAT], RKKRRQRRRP) conjugated to either scrambled (TAT-C_scram_, TC_sr_) or C-terminal with (TAT-Cter, TC) or without a Caax linker of CVIM (for membrane localization, TAT-Caax-Cter, TcxC) (Figure 6A). The CPPs successfully penetrated the cell membrane and were enriched in membrane regions, although a less fluorescence appeared to be in the scrambled TAT peptide presumably due to easier degradation of the scrambled CPPs inside of cells (Figure 6B). Next, we co-transfected cells with STrEP-TM4SF5 and the c-Src SH1 domain for 24 h, treated them with the CPPs for an additional 24 h, and then subjected them to co-immunoprecipitation. The interaction between TM4SF5 and the c-Src SH1 domain was blocked by the TcxC peptide but not by the control TC_sr_ peptide (Figure 6C). Furthermore, the enhanced phosphorylation of FAK, paxillin, c-Src, and p27^Kip1^ in TM4SF5-positive cells (SNU449_Tp_ cells) over control TM4SF5-lacking (SNU449_Cp_) cells 27, 29 was reduced more by the TC or TcxC peptides, as compared to TM4SF5-positive cells treated with the control TC_sr_ peptide (Figure 6D). This effect was also observed in other stable cell lines that ectopically expressed TM4SF5 (Figure S4A-B). However, treatment of parental SNU449 cells (lacking TM4SF5 expression) with either control TC_sr_, TC, or TcxC peptides did not affect signaling activities, compared with those of untreated cells (Figure S4C). In addition to the stable cells, HepG2 cells endogenously expressing TM4SF5 showed reduced phosphorylation of FAK, c-Src, and paxillin upon treatment with TC or TcxC, but not TC_sr_, peptide (Figure 6E). Furthermore, SNU398 cells with an insignificant TM4SF5 expression level showed minimal phosphorylation of FAK, c-Src, and paxillin which was not affected by CPP treatment (Figure 6F).

To determine whether another CPP sequence could mediate the same effects after conjugation to the TM4SF5 C-terminal sequence, the antennapedia homeodomain ([Antp] RQIKIWFQNRRMKWK) was conjugated to the scrambled or TM4SF5 C-terminal sequence with a linker sequence of CVIM (Figure S4D). Treatment of TM4SF5-expressing SNU449 (SNU449_Tp_) or SNU761 (SNU761 WT) cells with the Antp-Caax-Cter peptide resulted in inhibition of TM4SF5-dependent phosphorylation of FAK, paxillin, and c-Src unlike the control peptide (Figure S4E-F). Thus, the interaction between TM4SF5 and c-Src and downstream signaling activities were specifically abolished by two different CPPs containing the C-terminal sequence of TM4SF5.

### Cell-penetrating TM4SF5 C-terminal peptides blocked TM4SF5-mediated tumorigenesis and metastatic potential

**Figure 7:**
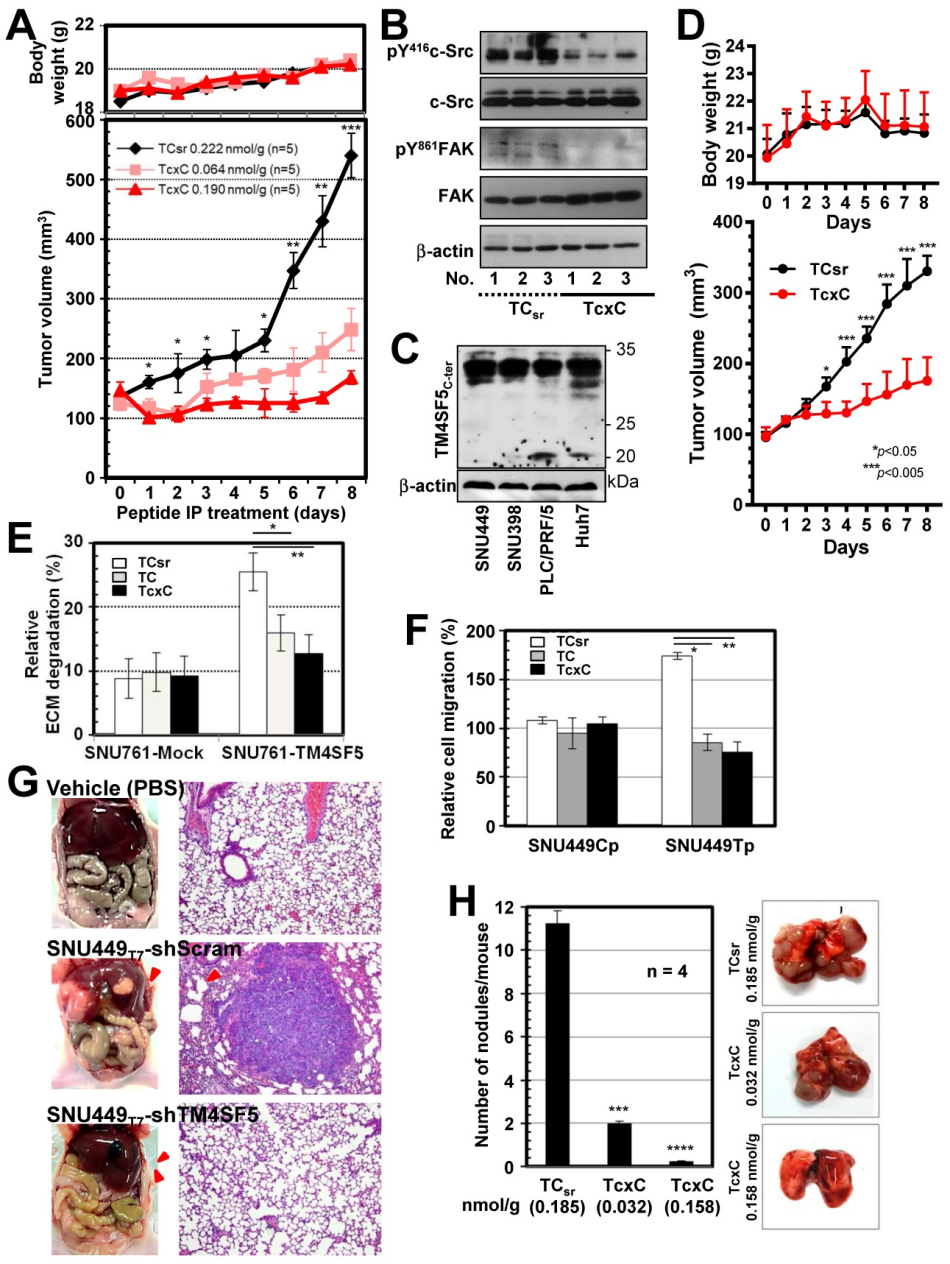
** TM4SF5 C-terminal peptides suppress TM4SF5-mediated tumorigenesis and metastasis.** (A and B) SNU449_T7_ cells stably expressing TM4SF5 were injected subcutaneously into nude mice (5 x 10^6^ cells/mouse, n =5). The cell-penetrating scramble or Caax peptides were administrated via intraperitoneal injections (at 0.222 nmol/g for TCsr and 0.064 or 0.190 nmol/g for TcxC) every day for 8 days, before tumor xenograft analysis, as explained in Materials and Methods. (B) Tissue extracts prepared from the xenograft tumors were processed to immunoblotting. (C and D) Different hepatoma cells including PLC/PRF/5 cells endogenously expressing TM4SF5, were immunoblotted for TM4SF5 expression (C). PLC/PRF/5 cells were injected subcutaneously into nude mice (1 x 10^7^ cells/mouse, n =6). The control TCsr or TcxC peptides were administrated via intraperitoneal injections (at 0.074 nmol/g for TCsr and 0.064 nmol/g for TcxC) every day for 8 days, before tumor xenograft analysis. (E) Degradation of Oregon Green® 488-conjugated gelatin were analyzed for SNU761 cells stably expressing mock (mock) or TM4SF5_WT_ with the 10 μM CPPs treatment for 24 h (mean ± SD values). (F) TM4SF5-null SNU449_Cp_ (Cp) and stably TM4SF5-expressing SNU449_Tp_ (Tp) cells were treated with the 10 μM CPPs for 24 h and then analyzed for transwell migration for 4 h (mean ± SD values in the graph). (G) Vehicle (PBS), SBU449_T7_ cells with infected with shRNA against either control sequence (shScram) or TM4SF5 (shTM4SF5) were once orthotopically injected to livers (n≥5) and 4 weeks later lung tissues were analyzed for the metastatic cell masses. (H) SNU449_T7_ cells were injected into the tail veins of mice (5 x 10^6^ cells/100 μl/mice, n=4). After 2 weeks, the CPPs were administrated at 0.037 or 0.185 nmol/g for TCsr and 0.032 or 0.158 nmol/g for TcxC every other day for 2 weeks. Two additional weeks later, animals were sacrificed for examination of *in vivo* lung metastasis. Representative lung images in each experimental condition are shown. Surface nodules were counted and the mean ± SD values are presented (graph). ANOVA with Tukey's range-test or two-tailed unpaired Student's *t*-test was done to determine the significance. NS depicts non-significance. *, **, ***, and **** depict *p* value less than 0.05, 0.01, 0.005, and 0.0001, respectively. The data represent three independent experiments.

To examine the effects of CPPs containing the TM4SF5 C-terminal sequence on TM4SF5-mediated tumor formation in nude mice, xenografts of SNU449_Tp_ cells were performed in the presence of CPP treatment by intraperitoneal (IP) injection every day for 8 days. Compared with the control CPP, which did not block tumor formation driven by SNU449_Tp_ cell injection, CPP containing the TM4SF5 C-terminal sequence inhibited tumor formation in a dose-dependent manner without any body weight loss (Figure 7A). Similar to CPPs containing the TM4SF5 C-terminal sequence, pharmacological inhibition of c-Src activity with PP2 suppressed TM4SF5-dependent tumor formation (Figure S5A). The inhibitory effects by the TcxC peptide in xenografts using TM4SF5-positive cells were consistent with a PP2-mediated suppressive effect (Fig. S5B). Further, whole tissue extracts from the xenografts showed lower pY^419^c-Src and pY^861^FAK levels upon TcxC treatment, compared with those that received TC_sr_ treatment (Figure 7B). In addition, we have examined whether xenografts by endogenously TM4SF5-expressing hepatoma cells were also affected by the peptides. Xenografts using endogenously TM4SF5-expressing hepatoma PLC/PRF/5 cells resulted in significantly less tumor volumes upon treatment of TcxC peptides, compared with those upon control TCsr peptide treatment (Fig. 7C-D).

We next examined whether TM4SF5-mediated metastatic potential could be targeted by the CPPs. Control SNU449_Cp_ or TM4SF5-expressing SNU449_Tp_ cells were treated with either CPP before immunostaining for pY^397^FAK or pY^861^FAK together with F-actin. CPP treatment of control cells did not affect focal adhesion formations as visualized by pY^397^FAK or pY^861^FAK, or stress fibers as visualized by phalloidin staining. Importantly, focal adhesions and stress fibers were reduced in SNU449_Tp_ cells treated with TM4SF5 C-terminus containing TC or TcxC CPPs, but not with the control TC_sr_ CPP (Figure S5C). The invasive ECM degradation and cell migration enhanced by TM4SF5 expression were also abolished by the TC or TcxC CPPs, but not by TC_sr_ CPP (Figure 7E-F).

We further examined TM4SF5-mediated metastatic function using animal models. We found that hepatic tumorigenesis and lung metastasis could be achieved via an orthotropic injection of TM4SF5-positive SNU449_Tp_ cells but not TM4SF5-suppressed SNY449_T7_-shTM4SF5 cells into the liver (Figure 7G). Next, we tested whether the TM4SF5-mediated lung metastasis of cells injected into tail veins could also be inhibited by the CPPs. The lung tissues showed that injection of TM4SF5-positive cells resulted in nodule formation, which was blocked by the TM4SF5 C-terminal CPPs in a dose-dependent manner, compared with those of control CPP-treated mice (Figure 7H). When the stability of the TcxC and TCsr peptides in mouse serum was examined, we found that 44-65% of the peptides remained after incubation for 24 h in the serum (Figure S6A). In addition, the peptides inhibited T cell activity (in terms of *IL-2* mRNA expression) when they were treated to differentiated Jurkat and My-La cells for 24 h, resulting in residual 59-63% activity without any effects on cell death (Figure S6B). The TcxC peptides may thus affect T cell activity, which is dependent on c-Src activity 52. The peptides may still be promising molecules for use in targeting TM4SF5 and c-Src in liver cancer. Thus, these *in vivo* observations provide clear support for the importance of TM4SF5 and c-Src interaction during hepatic tumorigenesis and progression, which can be blocked by CPPs containing the TM4SF5 C-terminal sequence.

## Discussion

**Figure 8:**
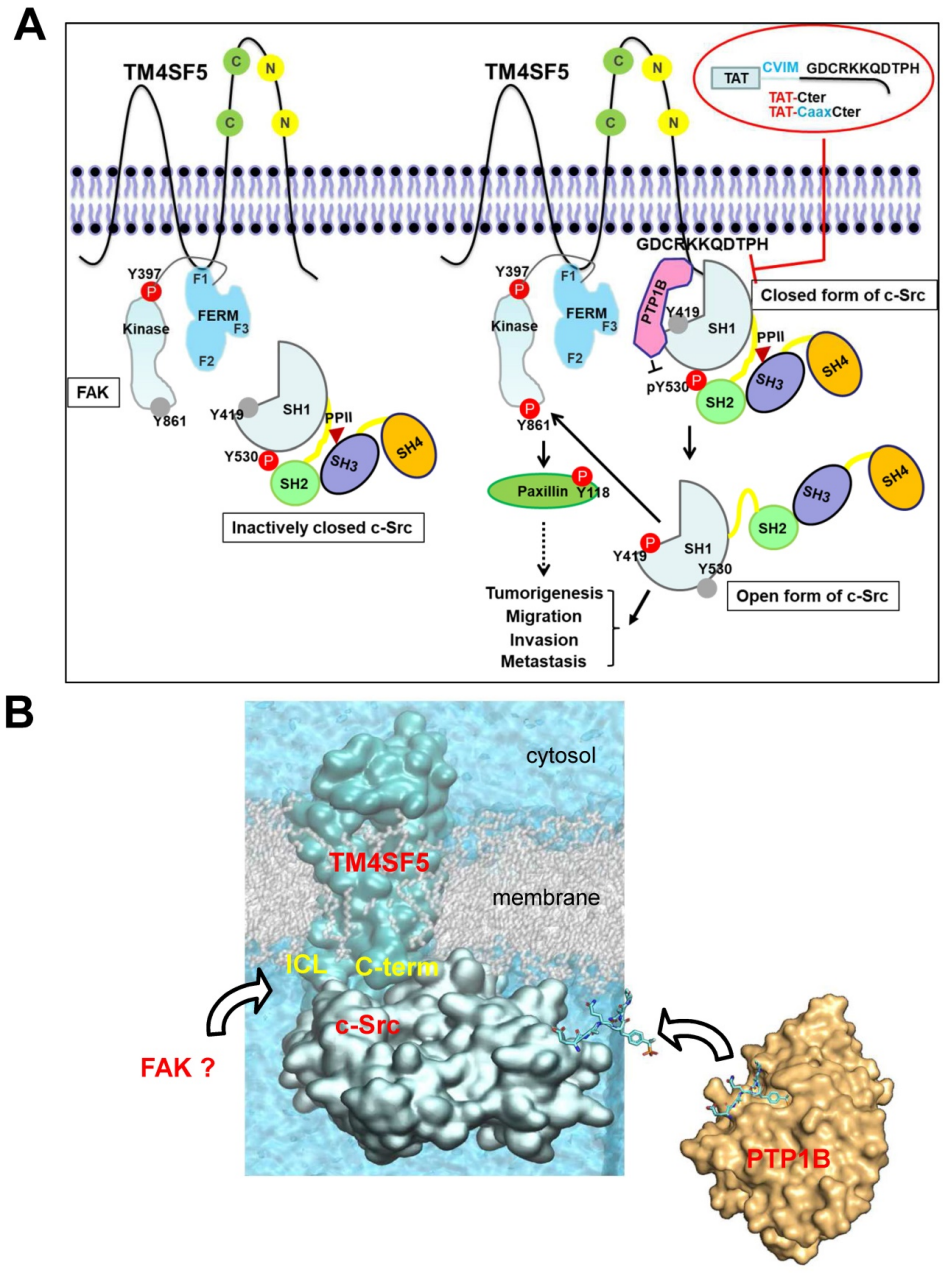
** The working model.** (A) TM4SF5, whose ICL interacts with the F1 lobe of FAK, prefers to directly bind to inactively-closed c-Src that is phosphorylated at the Y530 residue. After binding, TM4SF5 mediates dephosphorylation of phospho-Y530 by further recruiting PTP1B (i.e., unlatching). In addition, the TM4SF5 C-terminus-mediated interaction with the c-Src SH1 kinase domain may open its conformation (unclamping). Then the opened c-Src can be autophosphorylated at the Y419 residue (i.e., switching), leading to its full activation. Activated c-Src can phosphorylate Y861 of neighboring FAK. Activated c-Src and FAK then transduce downstream signaling pathways for TM4SF5-medated tumorigenesis and tumor progression. The CPPs containing the TM4SF5 C-terminal sequence interrupt the interaction between TM4SF5 and c-Src, suppress TM4SF5-mediated c-Src activation, and consequently inhibit TM4SF5-dependent tumorigenesis and metastatic potential. (B) Structural model of the membrane protein TM4SF5 complexed with c-Src. The plausible directions of the PTP1B and FAK bindings are depicted with black open arrows.

Proteins which bind the c-Src SH1 domain are rarely associated with its activation processes. However, in this study, we provide evidence that the C-terminal tail of membranous TM4SF5 binds the SH1 kinase domain of an inactive c-Src form with PTP1B, leading to N-terminus-independent activation of c-Src. The highly flexible nature of the cytosolic C-terminal tail of TM4SF5 seems to be responsible for the specific interaction to the c-Src kinase domain. Computational simulation, together with mutational confirmatory experiments, demonstrate how these floppy states work in the protein-protein interaction, offering new molecular-level insights on their roles in TM4SF5-mediated tumorigenesis and progression. Subsequent c-Src activation was possible by PTP1B-mediated dephosphorylation of c-Src Y530 via the triple complex formation, further leading to FAK Y861 phosphorylation. The interaction of PTP1B with TM4SF5 was not affected by a point mutation in the TM4SF5 C-terminus. Meanwhile, disruption of the LEL via C118A/C145A double mutation or treatment of TSAHC, which binds to the LEL 50, impaired TM4SF5 binding to c-Src. These data suggest a structural relay from the LEL to the cytosolic C-terminus for recruiting and activating c-Src. Association of the TM4SF5 C-terminus and c-Src kinase SH1 domain was disrupted by CPPs containing the TM4SF5 C-terminal sequence, which led to blockade of TM4SF5-mediated tumorigenesis and progression. Activating binders to the c-Src SH1 domain have not been identified yet. However, these observations suggest the existence of a signaling network involving the membrane protein TM4SF5, c-Src, and FAK resulting from unique interactions of the c-Src SH1 domain and PTP1B with TM4SF5 independent of the N-terminus of c-Src to mediate metastasis (Figure 8A-B). These molecular and structural aspects in TM4SF5-mediated c-Src activation can lead to insights in the development of therapeutic agents, including small compounds and peptides, against TM4SF5/c-Src-dependent carcinogenesis and progression.

TM4SF5 is highly expressed in a diversity of cancer cells and tissues in the liver 27 and other organs 53. TM4SF5 contains four transmembrane domains, SEL or LEL, and cytosolic N- and C-terminal tails 22. As a tetraspan(in), TM4SF5 can form TM4SF5-enriched microdomains (i.e., T_5_ERM) on the cell surface via protein-protein interactions to regulate cellular functions in a spatiotemporal manner 54. The LEL of TM4SF5 is known to be *N*-glycosylated and interacts with integrins 55, EGFR 56, CD44 57, or CD151 58 for migration and sphere growth, while the ICL binds FAK 24 for migration, and the C-terminus binds c-Src 25 for invasion. A membranous binder (i.e., TM4SF5) to the c-Src SH1 domain converts inactive c-Src to its active form. Indeed, the c-Src SH1 domain alone could be sufficient for binding to TM4SF5 and its activation level is comparable to that mediated by the SH321 domain (Figure 3G). This indicates that membranous TM4SF5 might compensate for mechanistic aspect(s) of c-Src activation, such as N-terminal myristoylation and ligand binding through the SH3 and SH2 domains 59. No other molecules have been shown to bind to the SH1 domain of c-Src besides β-arrestin1 60 and middle T-antigen 61. In contrast, the SH3 and SH2 domains have many binding partners, including Y397-phosphorylated FAK to mediate a diversity of cellular functions 62. β-arrestin1 binding to the c-Src SH1 domain regulates endocytosis of the β2-adrenergic receptor following ligand association and c-Src-mediated dynamin phosphorylation 60. During c-Src activation, relief of the inhibitory intramolecular interaction leads to an altered conformation around the activation loop of the SH1 kinase domain including Y419 7. Thus, TM4SF5 binding to the c-Src SH1 domain could be sufficient for relieving the intramolecular inhibitory interaction between the c-Src SH2 domain and phospho-Y530 following PTP1B-mediated dephosphorylation of Y530 (unlatching), leading to an open form (unclamping) and enhanced autophosphorylation at the Y419 residue (switching). Consistent with this, PTP1B is one of phosphatase that has been shown to dephosphorylate c-Src Y530 63. Meanwhile, interaction of the middle T-antigen with the SH1 domain also involves protein phosphatase 2A (PP2A) 61. The SH1 domain of c-Src alone (lacking the N-terminal lipid modification sites, SH4, SH3, and/or SH2 domains) led to phosphorylation of Tyr419 (Figure 2C). TM4SF5 binding to the c-Src SH1 domain could increase phospho-Y419 in this domain even without the positive roles induced by ligand binding to the SH3 and/or SH2 domains 59. The C-terminus of TM4SF5 bound more efficiently to inactive or closed c-Src than its active or open form even though active c-Src could still bind to TM4SF5. c-Src forms a dimer consisting of its active and inactive forms via the SH1 domain following Y419 phosphorylation as a prerequisite; however, this phosphorylation is dispensable once the dimer is formed 64. Thus, binding between non-receptor tyrosine kinase c-Src and membrane protein TM4SF5 could support the activation-dependent localization of c-Src to membranes, presumably independent of N-terminal lipid modification in c-Src 65.

Hepatocellular carcinoma (HCC) recurrence or metastasis appears to be critical for poor survival of HCC patients with TM4SF5 expression 50. Therapeutic trials to antagonize c-Src activity are actively being developed 66. Thus, targeting TM4SF5-dependent c-Src activity would be beneficial for the survival of HCC patients. Importantly, in this study, we demonstrate that CPPs including the TM4SF5 C-terminal sequence blocked TM4SF5-mediated c-Src activity. Although the CPPs with the TM4SF5 C-terminal sequences are relatively short, the introduction of peptide based-therapeutics to cells can be immunogenic, leading to the development of neutralizing antibody responses that could potentially limit this approach in the clinic. The CPP without the CVIM sequence (i.e., TC) was not evaluated for the inhibition of the binding of TM4SF5 to c-Src and the CVIM sequence itself might inhibit c-Src via another mechanism. However, the common antagonistic effects of TC and TcxC (but not TCsr) peptides against *in vitro* signaling activities depending on TM4SF5 expression could suggest that the interruption of the binding via the TM4SF5 C-terminus CPPs might lead to inhibitory roles in TM4SF5/c-Src-dependent cell functions. Meanwhile, TM4SF5 expression-mediated c-Src activity in hepatocytes leads to angiogenesis 67, invasive ECM degradation 25, and immune escape 68, suggesting that TM4SF5-mediated c-Src activation can play multiple roles during TM4SF5-mediated tumor progression 54. Further, administration of CPPs containing the TM4SF5 C-terminal sequence into mice injected with TM4SF5-expressing cells through the tail vein blocked TM4SF5-mediated tumor formation in the lung. Therefore, CPPs that disrupt the binding between TM4SF5 and c-Src can be therapeutically important for the treatment of TM4SF5-positive cancers.

## Supplementary Material

Supplementary figures and movie legends.

Supplementary movie 1.

Supplementary movie 2.
