# Supplementary material for: N-terminus-independent activation of c-Src via binding to a tetraspan(in) TM4SF5 in hepatocellular carcinoma is abolished by the TM4SF5 C-terminal peptide application
Source: Theranostics. 2021 Jul 6;11(16):8092–111. doi: 10.7150/thno.58739 (PMC8315060; doi:10.7150/thno.58739)
Supplement: Supplementary file 1 — Supplementary figures and movie legends. [file thnov11p8092s1.pdf]

N-terminus-independent activation of c-Src via binding to a tetraspan(in) TM4SF5 in hepatocellular carcinoma is abolished by the TM4SF5 C-terminal peptide application

Haeng Eun Song[1,2,†], Yoonji Lee[3,4,†], Eunmi Kim[1,2,†], Chang Yun Cho[1,2], Oisun Jung[1,2], Doohyung Lee[1,2], Eun Goo Lee[1,2], Seo Hee Nam[1,2], Minkyung Kang[1,2], Stephani Joy Y. Macalino[3], Ji Eon Kim[1,2], Jae Woo Jung[1,5], Sung Won Kwon[1,2], Sun Choi[3,*], and Jung Weon Lee[1,5,*]

Supplementary figures

Figure S1

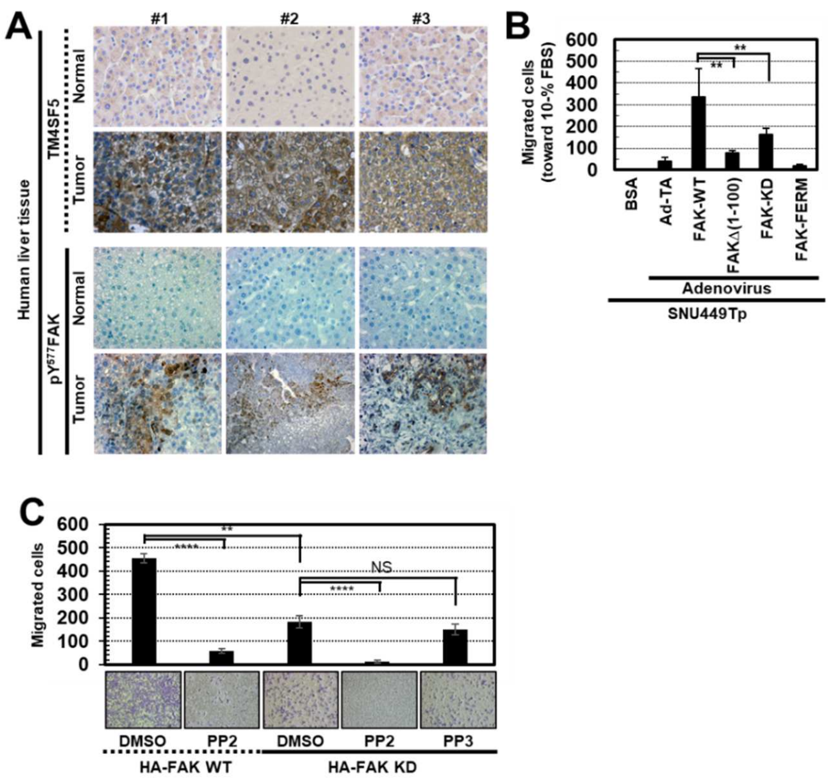

Figure S1. TM4SF5 involves tumorigenesis and migration supported by FAK and c-Src. (A) Human hepatic tumor tissues (n=3) were immunostained for TM4SF5 or phospho-Y577 FAK, in parallel with normal tissue from the same patients. (B and C) Cells were infected with adenovirus encoding for control or diverse FAK forms for 24 h, prior to transwell migration assay (10^5 cells/condition) for 12 h in the presence of 10% FBS-containing normal media in the lower chamber. PP2 or PP3 were treated at 10 μM at the time of cell seeding inside of the chamber. The error bars represent the mean ± standard deviation (SD). ANOVA with Tukey's range-test or two-tailed unpaired Student's *t*-test was done to determine the significance. NS depicts non-significance. *, **, ***, and **** depict p value less than 0.05, 0.01, 0.005, and 0.0001, respectively. The data represent three independent experiments.

Figure S2

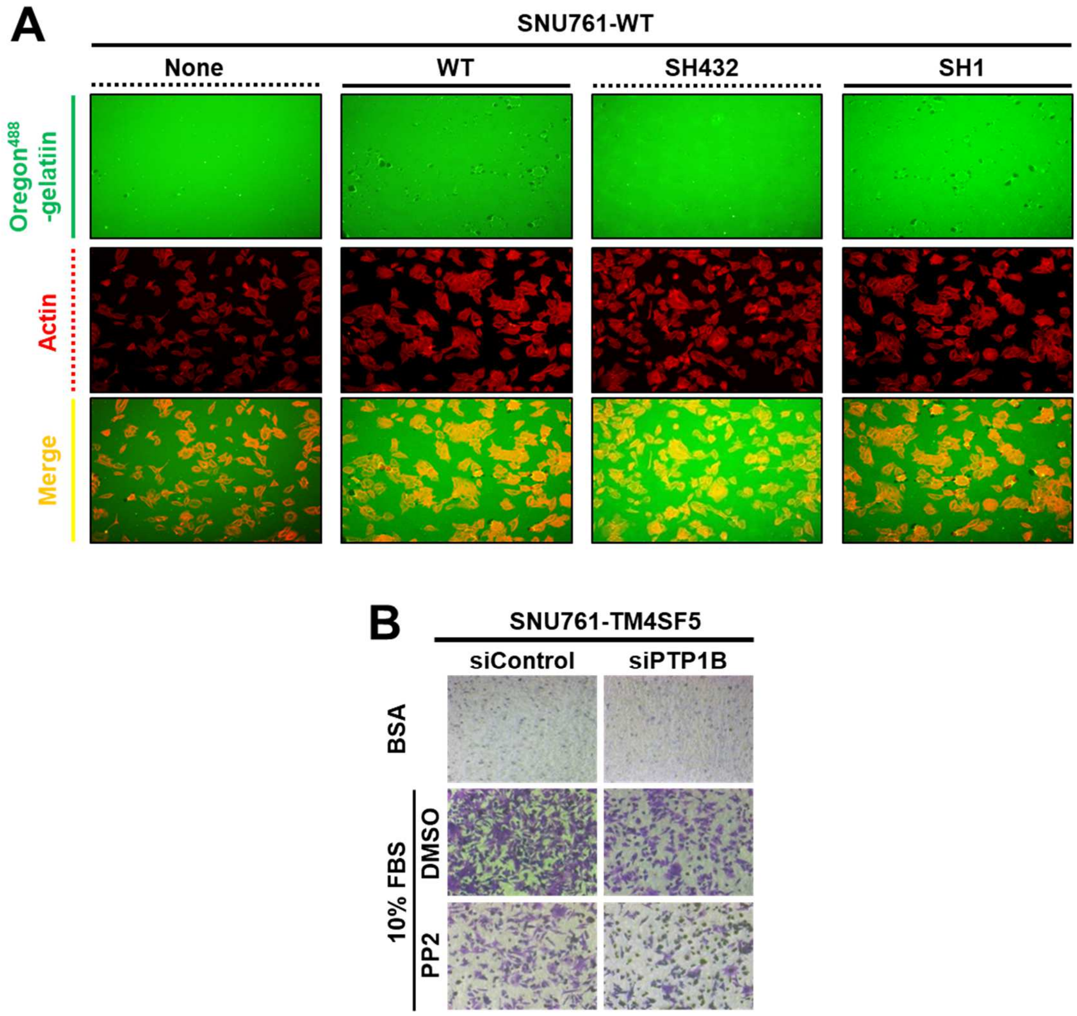

Figure S2. TM4SF5-mediated invasion and migration involves PTP1B-regulated c-Src activity. (A) SNU761 cells stably expressing mock or TM4SF5$_{WT}$ were transiently transfected without (No T/F) or with c-Src WT, c-Src SH432, or c-Src SH1 expression constructs for 48 h prior to invasive ECM degradation analysis for 4 h. (B) SNU761 cells stably expressing TM4SF5$_{WT}$ were transiently transfected with control siRNA against a scrambled sequence (siControl) or a sequence in PTP1B (siPTP1B) for 48 h, prior to transwell migration assay for 4 h. The media in the bottom chamber included 1% BSA alone (in serum free media) or normal 10% FBS containing media with DMSO or PP2 (10 μM). After incubation, migrated cells were randomly imaged for 10 fields per condition.

Figure S3

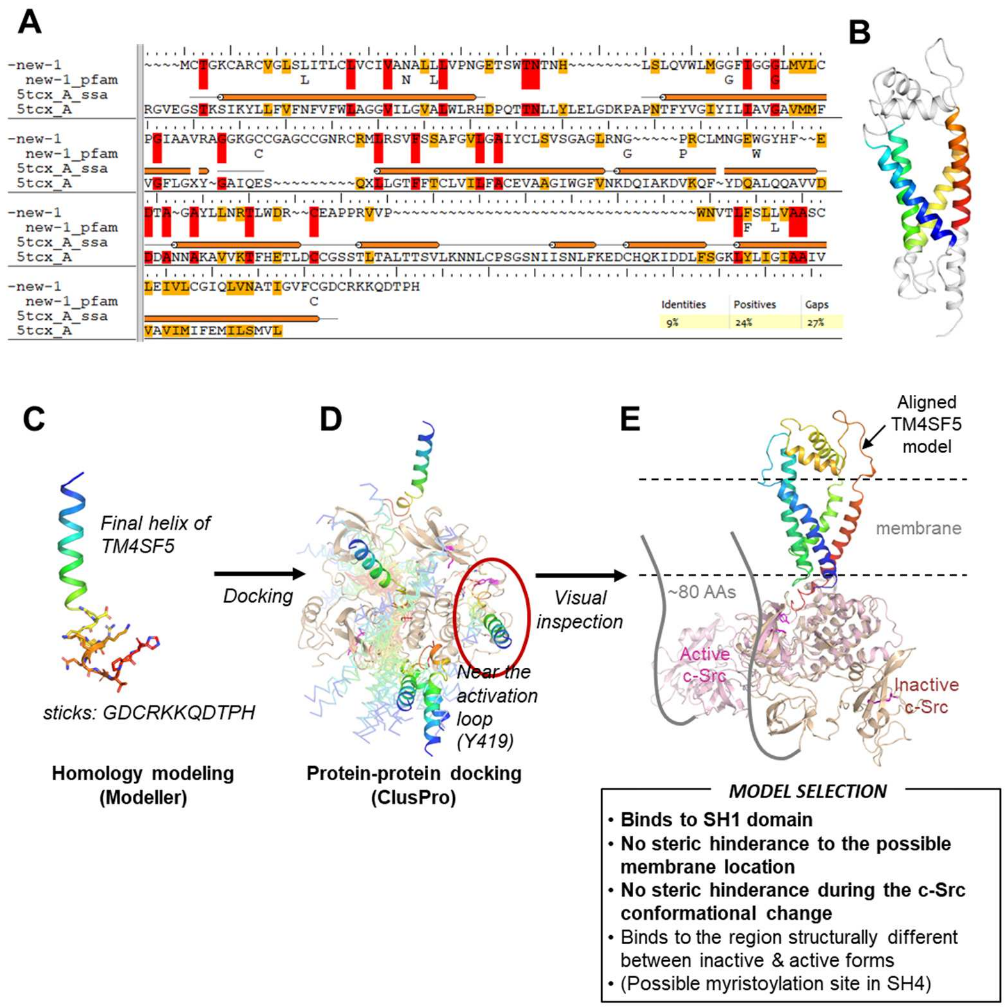

Figure S3. Molecular docking of the C-terminus of TM4SF5 to the SH1 domain of c-Src at an inactively-closed form. (A) The sequence alignment of TM4SF5 and CD81 (5TCX.pdb) that is utilized as a template for homology modeling. (B) The initial model of TM4SF5 generated by RosettaCM protocol. (C) Modeling of the TM4SF5 final helix (TM4) followed by the C-terminal tail. (D) Overlay of the generated models from ClusPro protein-protein docking. The docked TM4SF5 final helix along with C-terminal region is colored by blue-to-red (N-to-C termini). (E) The finally generated complex model. TM4SF5 is colored by blue-to-red (N-to-C termini). The inactive and active c-Src structures are colored in light yellow and pink, respectively. The missing residues (ca. 80 amino acids in this structure) at the N-terminal side of c-Src, including the myristoylation site for membrane targeting, are marked with gray curved lines.

Figure S4

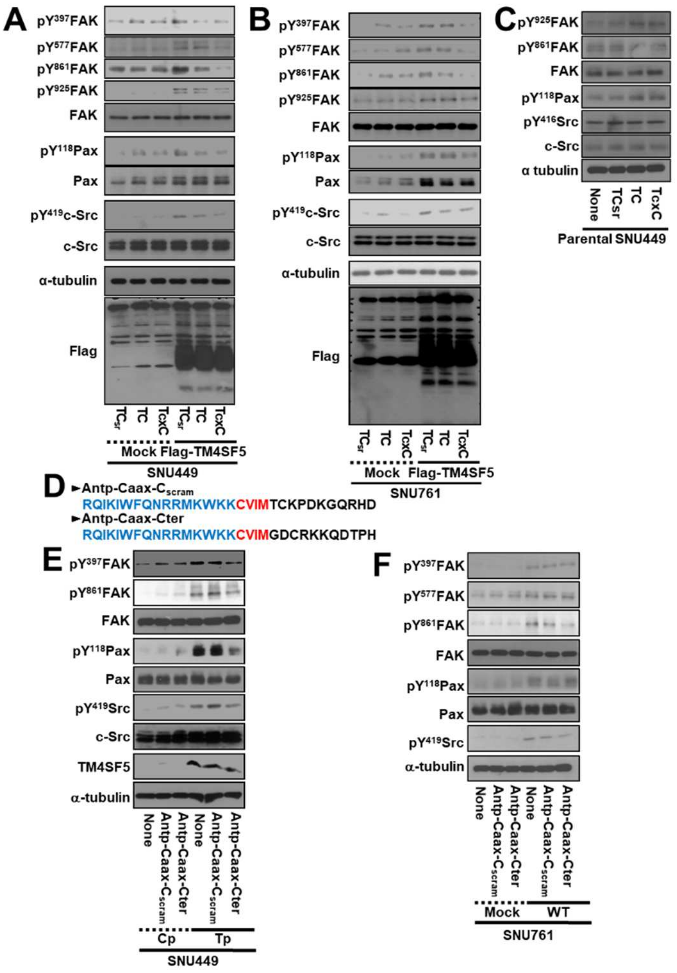

Figure S4. Antennapedia (Antp)-conjugated CPPs with the TM4SF5 C-terminal sequences blocks TM4SF5-dependent c-Src activation. (A-C) SNU449 (A) or SNU761 (B) cells stably transfected with mock or FLAG-TM4SF5 plasmids, in addition to parental SNU449 cells not expressing TM4SF5 (C) were treated without (none) or with the CPPs for 24 h, prior to harvests of whole cell lysates. Cell lysates were processed to the immunoblotting for the indicated molecules. (D) The sequences of the CPPs containing Antennapedia (Antp)-conjugated with a control (i.e., scrambled) sequence (Antp-Caax-C_{scram}), or the TM4SF5 C-terminal sequence with a Caax motif (Antp-Caax-Cter). (E-F) SNU449 (E) or SNU761 (F) cells stably-expressing mock (Cp or mock) or TM4SF5 (Tp or WT) were treated without (None) or with the CPPs for 24 h, prior to WCL harvests and immunoblotting for the indicated molecules. To clear comparisons among experimental conditions in TM4SF5-negative parental (C) or stable (E, Cp) SNU449 cells without any treatment, bands during the immunoblots were longer exposured at slightly higher levels (C), compared to those in non-treated SNU449Cp cells shown in (E). The data represent three independent experiments.

Figure S5

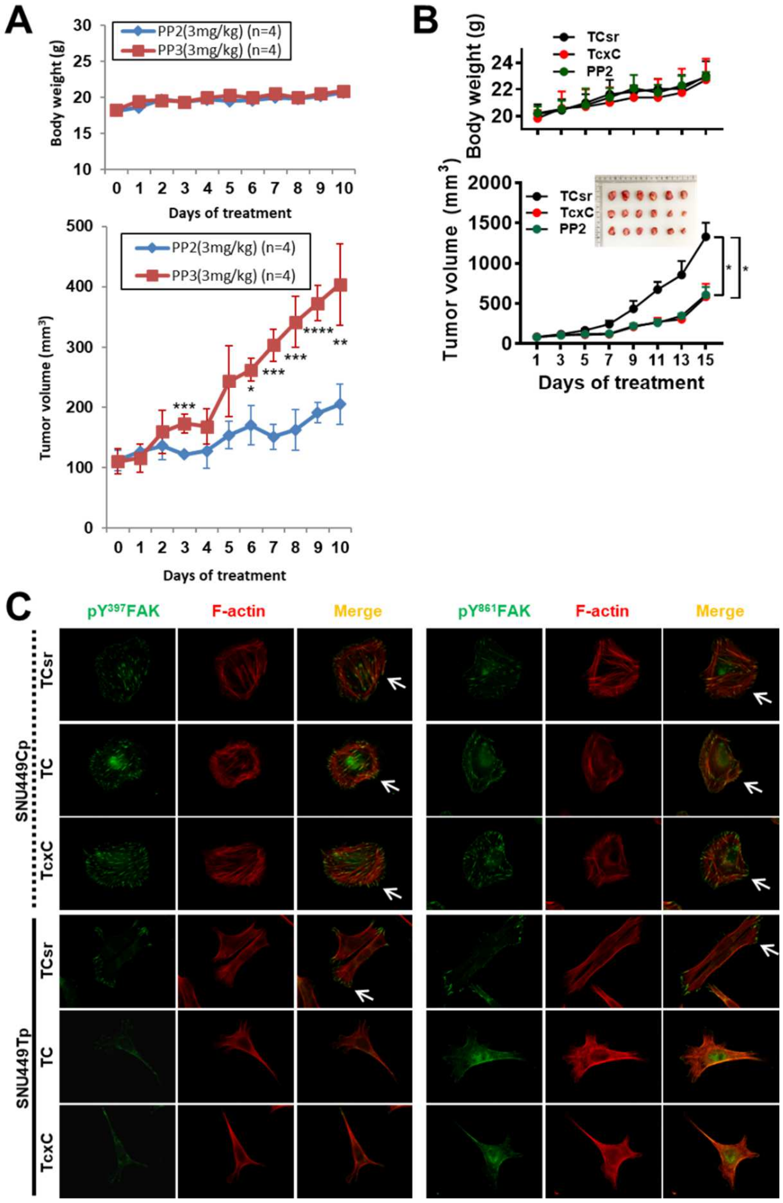

Figure S5. Physiological significance of inhibition of c-Src and treatment of CPPs with TM4SF5 C-terminus sequence in tumor growth and cellular morphology. (A-B) TM4SF5-expressing SNU449$_{T7}$ cells were subcutaneously injected at 5 x10^6 cells per BALB/c-nu/nu mice (n=4 for A and n=6 for B) and after the xenograft became 100 mm^3, tumor volumes and body weights were measured every day with treatment PP2 or PP3 at 3 mg/kg for 10 days (n =4, A) or with either PP2 (3 mg/kg), TCsr (0.111 nmol/g) or TcxC (0.095 nmol/g) peptides for 15 days (n=6, B). ANOVA with Tukey's range-test or two-tailed unpaired Student's *t*-test was done to determine the significance. *, **, ***, and **** depict p value less than 0.05, 0.01, 0.005, and 0.0001, respectively. (C) SNU449$_{Cp}$ control cells lacking TM4SF5 or SNU449$_{Tp}$ (Tp) cells stably expressing TM4SF5 were treated with the cell-penetrating TAT peptides for 24 h prior to indirect immunofluorescence for phospho-Y^{397}FAK or phospho-Y^{861}FAK (green) antibodies and staining with phalloidin (red). White arrows indicate focal adhesion spots.

Figure S6

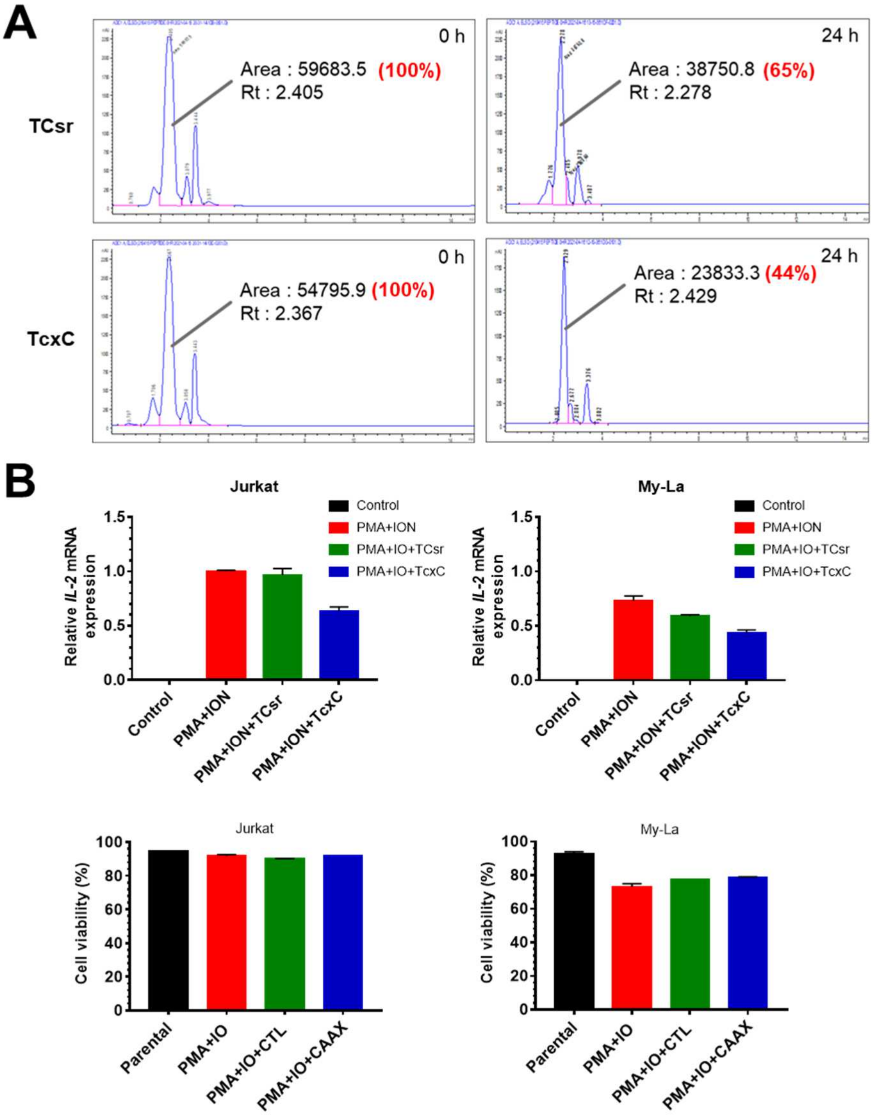

Figure S6. Stability of the TM4SF5 C-terminus CPP peptides and effects on T cell activity. (A) C57BL/6 mouse serum was prepared via centrifugation at 13,000 × *g* for 10 min and then separately mixed with the TCsr or TcxC peptides (at a final concentration of 10 ppm/reaction). At the incubation time point of 0 (without mixing with serum) or 24 h, the samples were mixed with 40 μL of 15% formic acid and incubated at 4℃ for 15 min to precipitate the high molecular weight proteins. The supernatant was collected after centrifugation at 13,000 × *g* for 10 min and stored at -20 ℃. To analyze, we used the ZORBAX Eclipse C18 Column (2.6 × 250 mm, 5 μm) in the Agilent 1200 Infinity LC and kept the column temperature at 40 ℃. Injection volume from samples was 20 μL and for the mobile phase, MS grade water with 0.1% formic acid (‘a’) and MS grade acetonitrile (‘b’) were flowed at the velocity of 1 mL/min. The gradient per sample was ‘a’ (5%)/’b’(95%) for 0 and 5 min, ‘a’ (90%)/’b’(10%) for 10 min, and ‘a’ (40%)/’b’(60%) for 15 min. Agilent 1260 infinity ELSD was used with an LC stack for detection. (B) Jurkat or My-La cells were treated without (control) or with phorbol 12-myristate 13-acetate (PMA, 10 nM) and ionomycin (1 μg/mL) for 24 h together with either TCsr or TcxC peptides at 1 nM. Then the cells were processed for qRT-PCR for relative *IL-2* mRNA expression analysis (top panel) or flow cytometry for cell death analysis following propidium iodide (PI) and annexin V staining (bottom panel). Data represent three independent experiments.

Supplementary movie spreadsheet

Movie S1. The overview of the optimization process for binding between TM4SF5 and c-Src. The simulated trajectories of three independent runs are shown together. The secondary structures of TM4SF5 and c-Src are colored in teal and light blue, respectively. The C-terminal tail of TM4SF5 that directly binds to c-Src is marked in red. The region of the lipid bilayer is displayed in gray dashed lines.

Movie S2. The detailed molecular interactions between the C-terminal tail of TM4SF5 and the c-Src kinase domain during the simulation. The secondary structures of TM4SF5 and c-Src are colored in teal and light blue, respectively. The C-terminal tail of TM4SF5 that directly binds to c-Src is marked in red. The amino acid residues in the C-terminal tail of TM4SF5 are represented in sticks with their carbon atoms in pink, and the interacting residues in c-Src are depicted in sticks with their carbon atoms in light blue. The ATP molecule bound in c-Src is displayed in ball-and-sticks.
